# Robust Prognostic Gene Expression Signatures in Bladder Cancer and Lung Adenocarcinoma Depend on Cell Cycle Related Genes

**DOI:** 10.1371/journal.pone.0085249

**Published:** 2014-01-22

**Authors:** Garrett M. Dancik, Dan Theodorescu

**Affiliations:** 1 Mathematics and Computer Science Department, Eastern Connecticut State University, Willimantic, Connecticut, United States of America; 2 Department of Surgery, University of Colorado, Aurora, Colorado, United States of America; 3 Department of Pharmacology, University of Colorado, Aurora, Colorado, United States of America; 4 University of Colorado Comprehensive Cancer Center, Aurora, Colorado, United States of America; Univesity of Texas Southwestern Medical Center at Dallas, United States of America

## Abstract

Few prognostic biomarkers are approved for clinical use primarily because their initial performance cannot be repeated in independent datasets. We posited that robust biomarkers could be obtained by identifying deregulated biological processes shared among tumor types having a common etiology. We performed a gene set enrichment analysis in 20 publicly available gene expression datasets comprising 1968 patients having one of the three most common tobacco-related cancers (lung, bladder, head and neck) and identified cell cycle related genes as the most consistently prognostic class of biomarkers in bladder (BL) and lung adenocarcinoma (LUAD). We also found the prognostic value of 13 of 14 published BL and LUAD signatures were dependent on cell cycle related genes, supporting the importance of cell cycle related biomarkers for prognosis. Interestingly, no prognostic gene classes were identified in squamous cell lung carcinoma or head and neck squamous cell carcinoma. Next, a specific 31 gene cell cycle proliferation (CCP) signature, previously derived in prostate tumors was evaluated and found predictive of outcome in BL and LUAD cohorts in univariate and multivariate analyses. Specifically, CCP score significantly enhanced the predictive ability of multivariate models based on standard clinical variables for progression in BL patients and survival in LUAD patients in multiple cohorts. We then generated random CCP signatures of various sizes and found sets of 10–15 genes had robust performance in these BL and LUAD cohorts, a finding that was confirmed in an independent cohort. Our work characterizes the importance of cell cycle related genes in prognostic signatures for BL and LUAD patients and identifies a specific signature likely to survive additional validation.

## Introduction

Gene expression profiling of human cancers has revolutionized our understanding of the disease and expedited the discovery of prognostic and predictive biomarkers [1]–[3]. However, few multi-gene biomarkers have been approved for clinical use [4] in part because they lack robustness across multiple datasets. This is especially striking for bladder cancer where Lauss and associates [5] evaluated 28 published gene signatures designed for diagnostic and prognostic purposes for bladder cancer and found that none of the 6 survival signatures performed better than chance when applied to independent datasets.

Here we address this lack of robust prognostic biomarkers by postulating the existence of common cellular processes (modules) across multiple tumor types of common etiology, whose abnormal activity could be captured by a transcriptional signature. As our goal was to develop robust bladder cancer biomarkers, we selected additional cancer types with smoking as a well-defined major etiological factor. The identification of such a process would presumably allow for development of robust biomarkers that are prognostic across multiple patient cohorts and tumor types.

**Figure 1:**
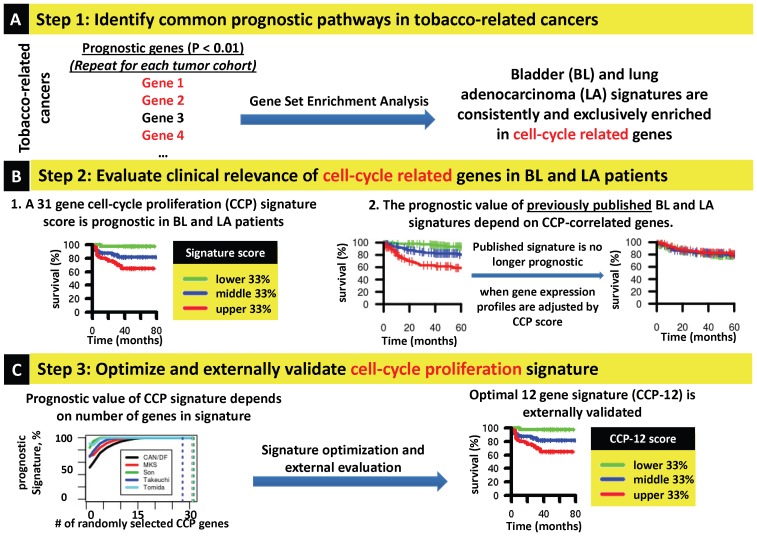
Study overview and summary of major findings. **A,** Biological processes associated with survival in tobacco-related tumors were identified through a gene set enrichment analysis. This analysis identified cell cycle as the only biological process consistently associated with outcome, in bladder and lung adenocarcinoma while no processes were identified that were predictive of outcome in lung adenocarcinoma, squamous cell lung carcinoma, or head and neck squamous cell carcinoma. **B,** Given the findings in **A,** the clinical relevance of cell cycle related genes was assessed in two ways. First, we evaluated the prognostic value of a specific 31 gene cell cycle proliferation (CCP) signature in bladder and lung adenocarcinoma in univariate and multivariate analysis. Second, we found that the prognostic value of previously published gene signatures predicting survival in bladder progression and lung adenocarcinoma was dependent on cell-cycle correlated genes. **C,** Because additional analysis revealed that the prognostic value of the CCP score was dependent on signature size, we optimized the CCP signature and found that a smaller 12 gene signature (CCP-12) was prognostic in an external dataset.

An overview of our analysis is provided in **Figure 1**. First, a gene set enrichment analysis of gene expression profiles from 1968 patients with tobacco-related cancers (bladder urothelial cell carcinoma, lung adenocarcinoma, lung squamous cell carcinoma, and head and neck squamous cell carcinoma) was performed and identified cycle related modules as consistently prognostic in bladder and lung adenocarcinoma. We found that a specific cell cycle gene signature was predictive of outcome in bladder and lung adenocarcinoma and that the performance of published bladder and lung adenocarcinoma signatures depended on cell cycle-correlated genes. Next we developed and evaluated a 12 gene panel of cell cycle related genes and found them effective in stratifying clinical outcome in an independent cohort. Our results characterize the core importance of cell cycle related biomarkers in prognostic gene signatures in patients with common cancer types and implicate cell proliferation as the primary driver of disease outcome. More broadly, this approach defines functionally dominant biological modules driving human cancer prognosis and identifies robust prognostic biomarkers.

## Materials and Methods

### Gene expression datasets and clinical endpoints

We analyzed 20 gene expression datasets comprising 1968 patients with bladder urothelial cell carcinoma (BL, five cohorts, 42*%*), lung adenocarcinoma (LUAD, eight cohorts, 39%), lung squamous cell carcinoma (LUSC, three cohorts, 8%), and head and neck squamous cell carcinoma (HNSCC, four cohorts, 10%). All gene expression datasets used in this analysis are publicly available and can be downloaded from the Gene Expression Omnibus (GEO) [6] or as supplemental material to publication, as indicated in **Tables S1–S4 in File S2**. References to the original study for each cohort are also provided in **Tables S1–S4 in File S2**. For more details see **Supporting Materials and Methods in File S1**.

Endpoints included progression in BL patients. Three BL cohorts had progression information, which was defined by the original authors as increase from NMI to MI disease (Lindgren and Dyrskjot cohorts, Ref # [7], [8]), or any increase in stage (CNUH, Ref # [9]). For two cohorts (Lindgren and CNUH) time to progression was not available and the ability of a gene or signature score to predict progression (progressor vs. non-progressor) was evaluated by area under the receiver operating characteristic curve (AUC). In Dyrskjot, clinical follow-up time was available allowing for progression-free survival (PFS) analysis.

The survival endpoint was selected as follows. Disease-specific survival (DSS) was always used if available (three BL cohorts). Overall survival (OS) was used if DSS was unavailable (two BL, seven LUAD, and two LUSC cohorts). Recurrence-free survival (RFS) was used if neither DSS nor OS were available (one LUAD and two HNSCC cohorts). The events for these endpoints are death from disease, death from any cause, and disease recurrence, for DSS, OS, and RFS, respectively. In one additional HNSCC cohort (Colo, Ref # [10]) recurrence status was provided but clinical follow-up times were not, and the ability of a gene to predict recurrence was evaluated by AUC. In addition, nodal involvement strongly correlates with outcome in HNSCC [11] and was used as a surrogate for survival in two cohorts allowing for a more comprehensive analysis. The clinical endpoints for each cohort are summarized in **Tables S1–S4 in File S2**.

### Cell cycle proliferation (CCP) score

In microarray datasets, probes were converted to gene symbols based on Affymetrix annotation, GenBank accession number, or Unigene cluster ID. For genes with multiple probes, the probe with the highest mean expression value was used [12]. The expression of each gene was *z*-normalized across samples to have a mean of zero and a standard deviation of one. CCP score is the average expression of all normalized CCP signature genes on the array. In the prostate cancer dataset, CCP score is the average expression of 31 CCP genes measured by quantitative RT-PCR. For details see **Supporting Materials and Methods in File S1**.

### Gene set enrichment and general statistical analyses

The Database for Visualization and Annotated Discovery (DAVID, Ref # [13]) was used for gene set enrichment analysis to identify overrepresented Gene Ontology (GO) [14] terms and KEGG pathways [15] in lists of genes. Additional details for this and general statistical analyses are provided in **Supporting Materials and Methods in File S1**. All analyses except for the gene set enrichment analysis was carried out using *R* (http://cran.r-project.org). Sample *R* code and output are provided in the form of a Sweave document that includes output from our analysis interweaved with corresponding *R* code (**File S3**). Additional R code is available upon request.

## Results

### Gene set enrichment analysis identifies cell cycle related genes as the most consistently prognostic class of biomarkers in bladder and lung adenocarcinoma

**Figure 2:**
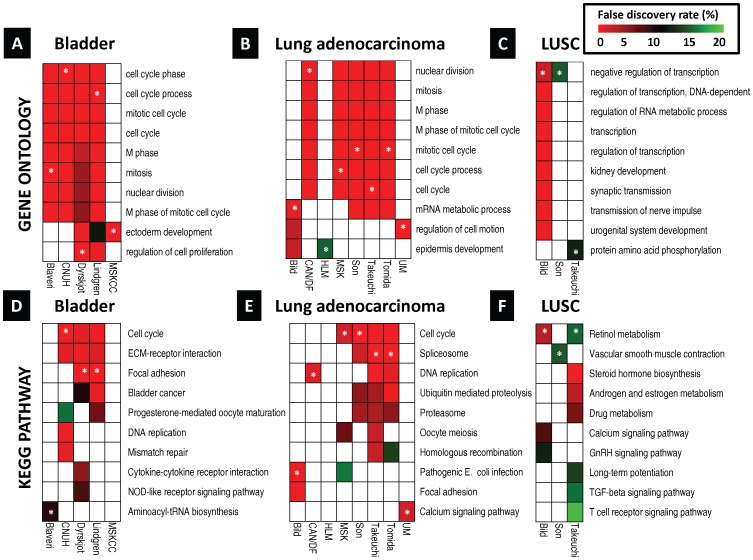
Prognostic modules associated with survival in tobacco-related cancers. In each cohort, over-represented Gene Ontology (GO) terms and KEGG pathways were identified from lists of genes significantly predictive of disease outcome (P<0.01) using the DAVID gene annotation enrichment analysis toolkit. Consistently prognostic modules were identified by ranking all modules first by the number of cohorts with significant results (FDR<20%) and then by average p-value. Each subfigure includes ten modules: the most consistently prognostic modules and the ‘top hit’ for each cohort, marked by an asterisk (*), which is defined as the module with the lowest FDR in that cohort that has an FDR<20% in multiple cohorts. **A,** over-represented GO terms associated with survival in bladder cancer. **B,** over-represented GO terms associated with survival in lung adenocarcinoma. **C,** over-represented GO terms associated with survival in squamous cell lung carcinoma. **D–F,** same as A–C except over-represented KEGG pathways are identified. There were no significantly over-represented prognostic modules in the head and neck squamous cell carcinoma cohorts at FDR<20%. LUSC: Squamous cell lung carcinoma, FDR: false discovery rate.

Data from 1968 patients were examined for the outcomes of tumor progression (where available) and survival (defined in **Materials and Methods**). In each cohort we identified lists of genes predictive of outcome (P<0.01) and performed an enrichment analysis that identifies overrepresented modules (GO terms [14] and KEGG pathways [15], **Figure 2** **, Figure S1,** and **Table S5 in File S6**) in these gene lists. Cell cycle related modules were the most consistently enriched modules across BL and LUAD patient cohorts (for details see **Supporting Results and Discussion in File S1**). In contrast, LUSC and HNSCC patient cohorts did not have a common overrepresented pathway (for details see **Supporting Results and Discussion in File S1**).

### Univariate and multivariate analysis of a cell cycle proliferation score in bladder and lung adenocarcinoma

To determine the clinical relevance of these findings we evaluated a previously published cell cycle proliferation (CCP) score (average expression of 31 cell cycle genes) that predicted time to recurrence or death in prostate cancer [16], [17]. If the overrepresented cell cycle modules were determinant of clinical outcome, then one would also expect CCP score to be.

**Figure 3:**
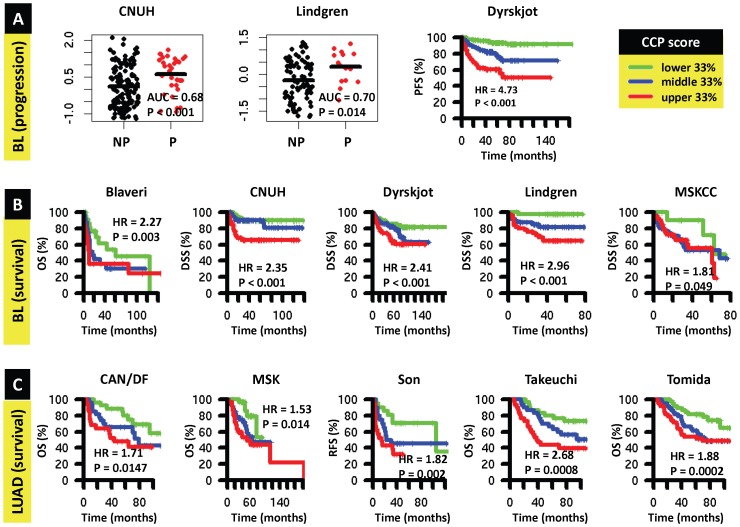
Prognostic value of cell cycle proliferation (CCP) gene score in bladder cancer and lung adenocarcinoma. **A,** prognostic value of CCP score for progression in bladder cancer in the CNUH (N = 165), Lindgren (N = 97), and Dyrskjot (N = 353) cohorts. In the CNUH and Lindgren cohorts, follow-up time was not available so we evaluated the ability of CCP score to discriminate between non-progressors (NP) and progressors (P) as described in **Materials and Methods**. In the Dyrskjot cohort, Kaplan-Meier (KM) curves for progression-free survival (PFS) were generated for patients with CCP scores at the lower (green), middle (blue), and upper (red) 33% and the log rank P-value of the continuous CCP score is reported. **B,** prognostic value of CCP score for survival in bladder cancer. KM curves were generated as in (A) for overall survival (OS) in the Blaveri (N = 74) cohort and for disease-specific survival (DSS) in the CNUH (N = 165), Dyrskjot (N = 366), Lindgren (N = 142), and MSKCC (N = 87) cohorts. **C,** prognostic value of CCP score for survival in lung adenocarcinoma. KM curves were generated for OS or recurrence-free survival (RFS) in the CAN/DF (N = 82), MSK (N = 104), Son (N = 62), Takeuchi (N = 90), and Tomida (N = 117) cohorts. Abbreviations: HR, hazard ratio, corresponding to 1-unit increase in CCP score.

Overall, CCP score was significantly predictive (P<0.05) of progression and survival in all BL cohorts with these endpoints, and of survival in 5/8 LUAD cohorts, with high CCP scores associated with poor prognosis in all cases. Specifically, CCP score was predictive of progression in CNUH (AUC = 0.68, P<0.05), Lindgren (AUC = 0.70, P<0.05), and Dyrskjot (HR = 4.73, P<0.001, **Figure 3A**) cohorts. CCP score was predictive of survival (P<0.05) in all five BL cohorts (HR 1.81–4.73, **Figure 3B**) CCP score was also predictive of outcome (P<0.05) in 5/8 LUAD cohorts (HR 1.53–2.68, **Figure 3C**).

We next evaluated whether CCP score contributes independent prognostic information. We performed a multivariate analysis and included clinically relevant variables such as age, gender, and grade in the BL and LUAD cohorts where CCP score was prognostic in the univariate analyses. For each cohort we also developed a best multivariate model (i.e., *final model*) through forward stepwise selection of informative variables (P<0.05). Such a model selects a concise, ‘optimal’ set of variables and may select CCP score over standard clinical variables. Because clinical variables are readily available this *final model* may not be cost-effective currently but may be so in settings where cancers are staged and graded according to their molecular pathology. Finally, we evaluated if CCP score increased the prognostic value of a *best available* model that included clinical variables that are readily available in current settings. Such an increase suggests that CCP score has clinical utility. For more details of the methodology see **Supporting Materials and Methods in File S1**.

CCP score was selected for the *final model* in all BL cohorts when progression was the endpoint (**Table 1**, **Table S6 in File S4**), and was the only significant variable in the multivariate analysis. When survival was the endpoint, CCP score was the most consistently significant variable in the multivariate analysis (P<0.05), along with stage, which were each significant in three cohorts. CCP score was selected for the *final model* in two of these cohorts (**Table 1**, **Table S7 in File S4**). In patients with LUAD, CCP score was the most consistently significant variable in the multivariate analysis and also the most frequently selected variable in the *final model* (**Table 2**, **Table S8 in File S4**). In the *best available* models, addition of CCP score led to an increase in prognostic ability (P<0.05) in two BL, one BL, and three LUAD cohorts when progression, survival, and survival were the endpoints, respectively (**Tables S9–S10 in File S5**). These results suggest that CCP score may be superior than standard clinical variables in both BL and LUAD cancers and that CCP score may have immediate clinical utility for predicting progression in BL patients and survival in LUAD patients. For more details see **Supporting Result and Discussion in File S1**. Consistent with the gene set enrichment analysis above, CCP score was not prognostic in patients with squamous tumors (LUSC and HNSCC) (**Figure S2**).

**Table 1:** Multivariate progression and survival analysis in patients with bladder cancer.

Dataset	Endpoint	Clinical variables*	Multivariate analysis (P<0.05)	Final model†
**CNUH (N = 165)**	Progression	CCP score, stage, grade, BCG therapy, chemotherapy, age, gender	**-**	**CCP score**, stage
**Lindgren (N = 97)**	Progression	CCP score, grade	**-**	**CCP score**
**Dyrskjot (N = 162)**	PFS	CCP score, stage, grade, CIS diagnosis, BCG/MMC treatment, age, gender	**CCP score**	**CCP score**
**Blaveri (N = 78)**	OS	CCP score, grade, stage, surgery, age, gender	**CCP score**	**CCP score**
**CNUH (N = 165)**	DSS	CCP score, stage, grade, BCG therapy, chemotherapy. age gender	Stage, age	Stage, age
**Dyrskjot (N = 155)**	DSS	CCP score, stage, grade, CIS diagnosis, cystectomy following TURBT, BCG/MMC treatment, age, gender	**CCP score**, CIS diagnosis	**CCP score**, CIS, age
**Lindgren (N = 156)**	DSS	CCP score, stage, grade, cystectomy following TURBT, age, gender	Stage	Stage
**MSKCC (N = 87)**	OS	CCP score, stage, grade, age, gender	**CCP score**, stage, grade	Stage, grade

* Variables include the following (see **Tables S6–S7 in File S4** for complete multivariate analysis):

Stage: Ta-T1 vs. T2–T4 (CNUH, Dyrksjot - DSS, Lindgren, and MSKCC) and T1 vs. Ta (Dyrskjot -PFS);

Grade: high vs. low (CNUH, Lindgren, Blaveri, MSKCC); high vs. low vs. PUNLMP (Dyrskjot).

Surgery: cystectomy vs. transurethral resection of the bladder.

Abbreviations: PFS, progression-free survival; OS, overall survival; DSS, disease-specific survival.

† Final model is constructed from forward step-wise regression of significant variables (P<0.05).

**Table 2:** Multivariate survival analysis in patients with lung adenocarcinoma.

Dataset	Clinical variables*	Multivariate analysis (P<0.05)	Final model†
**CANDF (N = 73)**	CCP score, stage, grade, chemotherapy, smoking history, age, gender	**CCP score**, chemotherapy, age	**CCP score**, chemotherapy, age
**MKS (N = 98)**	CCP score, stage, grade, radiotherapy treatment, chemotherapy, smoking history, age, gender	Grade	Grade, chemotherapy
**Takeuchi (N = 90)**	CCP score, stage, grade, EGFR status, KRAS status, p53 status, smoking history, age, gender	**CCP score**	**CCP score**, stage
**Tomida (N = 116)**	CCP score, stage, EGFR status, KRAS status, p53 status, smoking history, age, gender	**CCP score**	**CCP score**, stage

* Variables include the following (see **Table S8 in File S4** for complete multivariate analysis):

Stage: I vs. II (CANDF), I vs. II vs. III (MKS, Takeuchi, Tomida).

Grade: Well vs. moderately vs. poorly differentiated.

Smoking history: current/former vs. never-smoker.

EGFR, KRAS, and p53 status: mutant vs. wild-type.

† Final model is constructed from forward step-wise regression of significant variables (P<0.05).

### Prognostic signatures in bladder and lung carcinoma depend on cell cycle related genes

Because modules most consistently associated with progression in BL patients and survival in LUAD patients were cell cycle related (**Figure 2** **and Figure S1**), we hypothesized that the performance of previously defined BL progression and LUAD survival signatures would be dependent on cell cycle related genes. BL survival signatures were not examined because a previous study found that their performance was poor in independent datasets [18]. We tested our hypothesis on published gene signatures using an adjustment approach (for details see **Supporting Materials and Methods in File S1**) that attenuates the predictive capability of signature genes that are correlated with CCP score [19].

**Figure 4:**
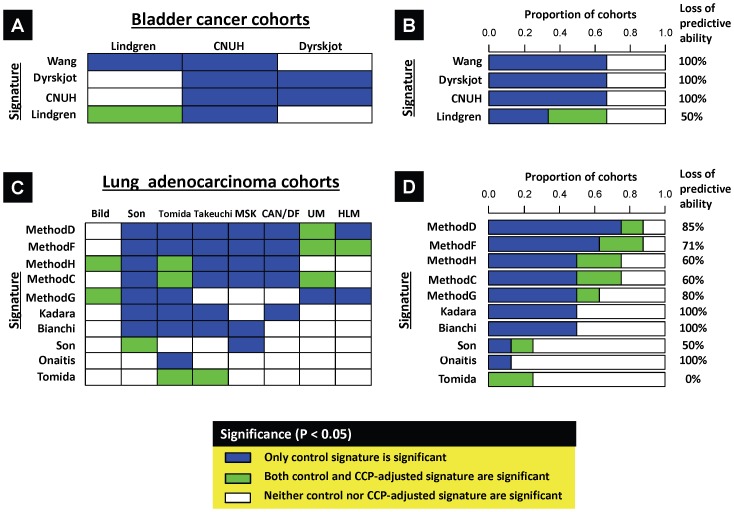
The prognostic value of published prognostic signatures in bladder cancer and lung adenocarcinoma. The expression values of prognostic signature genes were adjusted for CCP score or by a constant (negative control) as described in **Supporting Materials and Methods in File S1**. **A,** heatmap showing the impact of CCP score adjustment on the predictive ability of each signature (rows) on each cohort (columns). Signatures either lose their predictive ability following adjustment (P<0.05 in the control but P>0.05 in the CCP-adjusted cohort ; blue box); remain prognostic following the adjustment (P<0.05 in both the control and CCP-adjusted cohorts; green box); or were not prognostic in either case (P>0.05 in both groups, white box). **B,** stacked bar chart summarizing the prognostic value of each signature by categories described in (A). Loss of predictive ability is calculated as the percentage of signatures that lose their predictive ability following adjustment (blue boxes) with respect to the total number of control cohorts a signature is prognostic in (blue + green boxes). **C–D,** adjustment results in lung adenocarcinoma cohorts, in the same format as A–B.

In each dataset, expression levels of signature genes were adjusted for CCP score or by a constant term comprising a “negative control” (see **Supporting Materials and Methods in File S1** for details) and evaluated for progression in the BL cohorts and survival in the LUAD cohorts. A heatmap indicates each signature's prognostic value and whether its predictive ability is lost when adjusted for CCP score (**Figure 4**). In general, the signatures lost their predictive ability following CCP score adjustment. Specifically, bladder signatures lost their predictive ability in all independent cohorts, while lung signatures that were predictive in >2 cohorts lost their predictive ability in 83% of the cohorts they were predictive in, on average. For details see **Supporting Results and Discussion in File S1**.

### External validation of refined cell cycle expression signatures in human prostate cancer

Because CCP signature genes are highly correlated [16] and measure the same biological process, we hypothesized that a smaller gene signature would perform comparably to the full 31 gene signature. First, we characterized the importance of signature size through repeated random sampling of CCP genes to generate gene signatures of various sizes and assessed their prognostic value in the BL and LUAD cohorts. We found that the predictive power of CCP score depends on signature size and not necessarily gene composition, and that 10–15 genes are sufficient to maintain the prognostic power of the full signature (**Figures S3, S4**) (see **Supporting Results and Discussion in File S1** for details). To independently confirm this finding and to evaluate smaller refined signatures we analyzed 4 “robust” signatures (CCP-4, -7, -10, and -12) (**Table S12 in File S8**) which include genes predictive of outcome in multiple cohorts (see **Supporting Materials and Methods in File S1** for details).

The CCP-4, -7, -10, and -12 signatures were provided to Myriad Genetics (Salt Lake City, UT) for “blinded” evaluation, i.e., without indication of our hypothesis regarding the relationship between signature size and prognostic value. The predictive ability of the signature scores for biochemical recurrence in prostate cancer was evaluated in 353 patients receiving radical prostatectomy [17], with gene expression measured by quantitative RT-PCR (see **Supporting Materials and Methods in File S1**). All scores were predictive (P<0.05) in univariate and multivariate analyses which included CAPRA-S [20], a measure of clinical risk accounting for pre-operative PSA, pathologic Gleason score, surgical margins, extra-capsular extension, seminal vesicle invasion, and lymph node invasion. In general, hazard ratios increased and p-values decreased with increasing gene number (**Table 3**). The refined signature scores were then analyzed in a multivariate analysis testing whether the full signature added predictive value to a model that also included CAPRA-S. The full signature added significant predictive value (P<0.05) to the CCP-4, CCP-7, and CCP-10 models, but not to the CCP-12 model, defining this signature (BIRC5, BUB1B, CDC20, CDCA8, CENPF, FOXM1, KIF11, NUSAP1, PTTG1, TK1, TOP2A) as sufficient.

**Table 3:** Analysis of refined CCP signatures in prostate cancer.

CCP score	Univariate HR (95% CI)	Univariate P-value	P-value adjusted by CAPRA-S	P-value for CCP- 31 adjusted by refined CCP score and CAPRA-S
**CCP-31**	1.99 (1.61, 2.45)	2.00E-09	7.60E-07	-
**CCP-12**	1.96 (1.59, 2.41)	1.70E-09	7.20E-07	0.61
**CCP-10**	1.87 (1.52, 2.30)	3.10E-08	5.10E-06	0.032
**CCP-7**	1.87 (1.50, 2.32)	1.00E-07	5.60E-06	0.033
**CCP-4**	1.69 (1.37, 2.08)	2.30E-06	3.90E-05	0.0037

**Figure 5:**
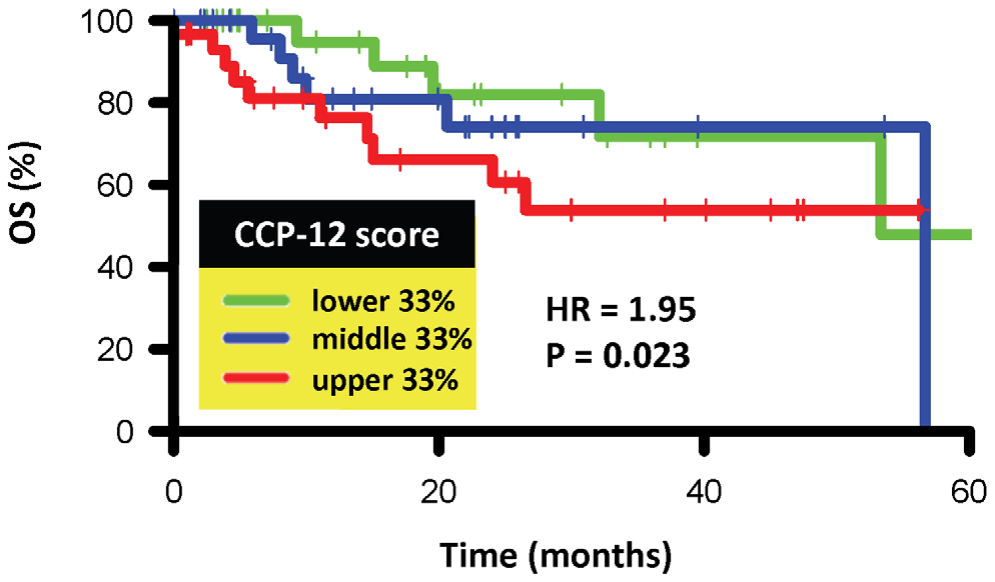
Prognostic value of a refined 12-gene cell cycle proliferation (CCP-12) score. Prognostic value of a refined 12-gene cell cycle proliferation (CCP-12) score in lung adenocarcinoma patients with gene expression profiling by RNASeq is shown. Kaplan-Meier (KM) curves for overall survival (OS) were generated for patients (N = 88) with CCP-12 scores at the lower (green), middle (blue), and upper (red) 33% and the log rank P-value of the continuous CCP-12 score is reported. Abbreviations: HR, hazard ratio, corresponding to 1-unit increase in CCP-12 score.

Finally, we evaluated the CCP-12 signature in an additional cohort consisting of 88 patients with lung adenocarcinoma of mixed subtypes. Gene expression profiles for these patients were obtained through RNA sequencing (RNASeq) and downloaded from The Cancer Genome Atlas (TCGA) (http://cancergenome.nih.gov). CCP score was calculated as before (the average row-normalized expression of the 12 CCP genes) using the normalized RNA counts (RNAseq by Expectation-Maximization, [21]) available from TCGA. **Fig. 5** shows that CCP-12 significantly predicts OS in these patients (HR = 1.95, P = 0.023), confirming the predictive ability of CCP-12 in an external cohort and indicating that CCP score is robust across multiple gene expression profiling technologies (microarray and RNAseq).

## Discussion

We performed a gene set enrichment analysis of gene expression profiles in patients with four tobacco-related cancers and identified cell cycle as the functional process most consistently associated with patient outcome in tumors with non-squamous histologies. Aberrant cell proliferation is a hallmark of cancer [22] and increased expression of cell cycle genes is found in multiple tumor types [23], including those not causally associated with smoking such as breast [19] and prostate cancer [16], [17]. However, it is interesting to note that smokers with prostate cancer have an increased risk of recurrence and mortality [24]. Surprisingly, cell cycle associated genes were not prognostic in tobacco-related tumors with squamous histology. However, we note these cohorts had relatively few patients (<85) which may have precluded the identification of functional processes associated with outcome in these tumor types

Our data provides important insights beyond the identification of a specific prognostic signature (CCP-12). First, it suggests that in BL and LUAD cancers, prognostic biomarkers will not validate across multiple cohorts unless they are (directly or indirectly) associated with cell cycle (**Figure 4**). Second, our finding that gene expression signatures could predict survival in multiple bladder cancer cohorts was unexpected because a recent validation study found that multiple signatures [25], [26] derived from datasets examined here (Blaveri, MSKCC) had poor predictive ability [18]. Third, our data demonstrates for the first time that robust survival gene expression signatures exist in bladder cancer but only when defined based on functional gene modules. If used on other tumor types, this concept may lead to the development of robust signatures that are more likely to reach clinical practice.

Our work also tackles an important but seldom addressed issue in biomarker development, the impact of signature size on predictive ability. Cost effectiveness and feasibility of assessing multiple genes in a small quantity of biopsy tissue weigh against the need for large enough signatures that overcome technical variability from the assay and biological variability from tumor sample heterogeneity. Our results indicate the prognostic performance of cell cycle genes plateaus at 10–15. In contrast, Haibe-Kains *et. al*. found that in breast cancer the prognostic value of AURKA expression was comparable to multi-gene models. AURKA is one of the cell cycle genes we examined but was not as robust as CCP score, being predictive (P<0.05) in only 5/13 BL and LUAD cohorts (**Figure S5**). In general, redundancy obtained from multiple genes may overcome the variable quality of RNA derived from fixed tissue, which can cause individual gene assays to fail, thus ensuring robust analytical results. While we found that CCP-12 predicted outcome in an independent cohort additional steps such as defining thresholds that would separate patients into high and low risk groups and prospective evaluation in independent cohorts using predefined endpoints are required before this panel is ready for clinical use.

## Supporting Information

File S1 **Supporting Materials, Results and Discussion.**
(DOCX)

File S2 **Summary of bladder cancer (Table S1), lung adenocarcinoma (Table S2), lung squamous cell carcinoma (Table S3), and head and neck squamous cell carcinoma (Table S4) patient cohorts.**
(PDF)

File S3 **Sweave document containing sample** ***R*** **code and output.**
(PDF)

File S4 **Multivariate analyses of progression in bladder cancer (Table S6), survival in bladder cancer (Table S7), and survival in lung adenocarcinoma (Table S8).**
(PDF)

File S5 **Comparison of prognostic power of CCP score and best available clinical variables in bladder (Table S9) and lung adenocarcinoma (Table S10).**
(PDF)

File S6 **Prognostic modules associated with outcome in tobacco-related cancers (Table S5) (Excel XLSX file).** In each cohort, over-represented Gene Ontology (GO) terms and KEGG pathways were identified from lists of genes significantly predictive of disease outcome (progression in BL patients and survival in BL, LUAD, LUSC, and HNSCC patients, P<0.01) using the DAVID gene annotation enrichment analysis toolkit. Consistently prognostic modules were identified by ranking all modules first by the number of cohorts with significant results (FDR<20%) and then by average p-value. There were no significantly over-represented prognostic modules in HNSCC patient cohorts at FDR<20%.
(XLS)

File S7 **Prognostic bladder progression and lung adenocarcinoma survival gene signatures used in the adjustment analysis (Table S11) (Excel XLSX file).**
(XLS)

File S8 **CCP genes in five refined signatures (Table S12).** Signatures are denoted by the number of CCP genes.
(DOCX)

File S9 **Prognostic modules associated with bladder progression and lung adenocarcinoma survival signatures (Table S13) (Excel XLSX file).** Over-represented Gene Ontology (GO) terms and KEGG pathways were identified for each signature using the DAVID gene annotation enrichment analysis toolkit. Modules are ranked first by the number of signatures with significant results (FDR<20%) and then by average p-value.
(XLSX)

Figure S1 **Prognostic modules associated with progression in bladder cancer.** In each cohort, over-represented Gene Ontology (GO) terms and KEGG pathways were identified from lists of genes significantly predictive of progression (P<0.01) using the DAVID gene annotation enrichment analysis toolkit. Consistently prognostic modules were identified by ranking all modules first by the number of cohorts with significant results (FDR<20%) and then by average p-value. Each subfigure includes ten modules: the most consistently prognostic modules and the ‘top hit’ for each cohort, marked by an asterisk (*), which is defined as the module with the lowest false discovery rate (FDR) in that cohort that has an FDR<20% in multiple cohorts. **A,** over-represented GO terms associated with progression in bladder cancer. **B,** over-represented KEGG pathways associated with progression in bladder cancer.
(TIF)

Figure S2 **Prognostic value of CCP score in squamous cell lung cancers and head and neck squamous cell carcinomas.** **A,** prognostic value of CCP score in squamous cell lung carcinomas (SCLC). Kaplan-Meier (KM) curves were generated for overall survival (OS) in the Bild (N = 53) and Takeuchi (N = 35) cohorts and for recurrence-free survival (RFS) in the Son (N = 76) cohort. KM curves were generated for patients with CCP scores at the lower (green), middle (blue), and upper (red) 33% and the log rank P-value of the continuous CCP score is reported. **B,** prognostic value of CCP score in head and neck squamous cell carcinomas (HNSCC). The Colo (N = 81) cohort did not include clinical follow-up time and so we evaluated the ability of CCP score to discriminate between node negative (N0) and node positive (N+) patients or between patients with non-recurrent (NR) and recurrent (R) tumors. The Pavon cohort (N = 63) did not include any clinical endpoints and so we evaluated the ability of CCP score to discriminate between N0 and N+ patients. In the Cohen cohort (N = 44), KM curves were generated for RFS. Abbreviations: HR, hazard ratio, corresponding to 1-unit increase in CCP score.
(TIF)

Figure S3 **Relationship between prognostic value of CCP score and signature size based on proportion of significant signatures.** Up to 10,000 gene signatures of sizes 1, 2, 4, …30, 31 were generated as described in **Supporting Materials and Methods in File S1**. Solid lines indicate proportion of signatures at each size that predicted survival (P<0.05) and are colored according to **A,** bladder patient cohort and **B,** lung adenocarcinoma cohort. Vertical dotted lines correspond to number of CCP genes (of 31) profiled in each cohort and are colored according to cohort.
(TIF)

Figure S4 **Relationship between prognostic value of CCP score and signature size based on p-values.** Up to 10,000 gene signatures of sizes 1, 2, 4, …30, 31 were generated as described in **Supporting Materials and Methods in File S1**. Boxplots of log10 p-values of signature scores for each signature size are plotted in **A,** bladder patient cohorts and **B,** lung adenocarcinoma cohorts. The blue horizontal line corresponds to a p-value of 0.05.
(TIF)

Figure S5 **Prognostic value of CCP signature genes** in **A,** bladder cancer and **B,** lung adenocarcinoma cohorts. In each cohort a gene is either significantly predictive of outcome (red box, P<0.05), not significantly predictive of outcome (gray box, P≥0.05), or was not profiled (white box) in each cohort. * indicates CCP score (using all available genes) is prognostic (P<0.05).
(TIF)
