# Supplementary material for: Robust Prognostic Gene Expression Signatures in Bladder Cancer and Lung Adenocarcinoma Depend on Cell Cycle Related Genes
Source: PLoS One. 2014 Jan 22;9(1):e85249. doi: 10.1371/journal.pone.0085249 (PMC3898982; doi:10.1371/journal.pone.0085249)
Supplement: File S1 — Supporting Materials, Results and Discussion. (DOCX) [file pone.0085249.s001.docx]

**PONE-D-13-16641R3 - SUPPORTING INFORMATION**

**Robust prognostic gene expression signatures in bladder cancer and lung adenocarcinoma depend on cell cycle related genes**

Garrett M. Dancik^1^ and Dan Theodorescu ^2,3,4,*^

**^1^**Mathematics and Computer Science Department, Eastern Connecticut State University, Willimantic, Connecticut, 06226 USA

^2^ Department of Surgery, University of Colorado, Aurora, Colorado, 80045 USA

^3^ Department of Pharmacology, University of Colorado, Aurora, Colorado, 80045

^4^ University of Colorado Comprehensive Cancer Center, Aurora, Colorado, 80045

^*^Corresponding author

**SUPPORTING MATERIALS AND METHODS**

**Gene expression datasets**

We analyzed 20 gene expression datasets comprising 1968 patients with bladder urothelial cell carcinoma (BL, five cohorts, 42*%*), lung adenocarcinoma (LUAD, eight cohorts, 39%), lung squamous cell carcinoma (LUSC, three cohorts, 8%), and head and neck squamous cell carcinoma (HNSCC, four cohorts, 10%). All gene expression datasets used in this analysis are publicly available and can be downloaded from the Gene Expression Omnibus (GEO) [[1](#_ENREF_1)] or as supplemental material to publication (**Tables S1-S4 in File S2)**. Lung, bladder and head and neck gene expression datasets were identified by searching GEO [[1](#_ENREF_1)] and Array Express databases [[2](#_ENREF_2)], as well as the literature. We analyzed all BL cohorts with more than 30 samples with clinical outcome information (survival or progression) that were deposited on GEO or Array Express and also obtained two additional published datasets (MSKCC and Blaveri) [[3](#_ENREF_3),[4](#_ENREF_4)] available as supplementary material to publication. We analyzed all lung cohorts with ≥30 LUAD or LUSC samples and clinical outcome information (survival, progression, or recurrence). We downloaded data from the six available HNSCC cohorts. However, two cohorts [[5](#_ENREF_5),[6](#_ENREF_6)] did not contain any outcome information and were not analyzed while another cohort [[7](#_ENREF_7)] did not contain any CCP signature genes and was used only in the functional analysis.

**Data processing**

We used the processed data for the bladder, head and neck, and lung cancer microarray datasets (**Tables S1-S4 in File S2)**. Two cohorts (Blaveri, Tomida) contained missing values. Probes with missing values in >20% of samples were removed and expression values imputed using the *impute* package (*impute.knn* function) in *R* with default parameters [[8](#_ENREF_8)]. Replicate samples in the Dyrskjot cohort were averaged to produce a single gene expression profile for each patient. The prostate cancer dataset consisted of gene expression profiles obtained from RT-PCR and is described in [[9](#_ENREF_9)].

**Gene signatures**

The cell cycle proliferation (CCP) signature [[9](#_ENREF_9)], bladder cancer progression signatures [[10](#_ENREF_10),[11](#_ENREF_11),[12](#_ENREF_12),[13](#_ENREF_13)], and lung adenocarcinoma signatures [[11](#_ENREF_11),[14](#_ENREF_14),[15](#_ENREF_15),[16](#_ENREF_16),[17](#_ENREF_17),[18](#_ENREF_18)] were obtained from the literature and gene symbols updated using the Hugo Gene Nomenclature Committee (HGNC) website [[19](#_ENREF_19)]. The updated gene signatures are provided in **Table S11**.

**Evaluation of gene signatures adjusted by cell cycle proliferation score**

For each gene signature, adjustment was carried out at the probe level using all relevant probes. Gene expression of probes was adjusted for cell cycle proliferation (CCP) score as follows: for each probe in the signature, we fit the linear model$y=\beta_{0}+\beta_{1}s$, where $y$is a vector of expression values and $s$ is a vector of corresponding CCP scores. For each probe, its expression vector $y$ is replaced by the residual vector where $\hat{\beta}_{0}$and $\hat{\beta}_{1}$ are the parameter estimates from the linear regression model [[20](#_ENREF_20)]. After the adjustment, a predictive probe that is correlated with CCP score will lose its predictive ability, while a predictive probe that is *not* correlated with CCP score will remain predictive. In this way, we can evaluate whether the prognostic value of a gene signature is driven by CCP-correlated genes. Adjusted gene expression profiles are compared to unadjusted controls calculated as described above but using a linear model with only an intercept term (i.e., we set$\beta_{1}\equiv0$). The predictive ability of adjusted and control gene expression profiles were evaluated by leave-one-out cross validation using a nearest centroid classification algorithm (the *pamr* package in *R* with a threshold of zero) [[21](#_ENREF_21)].

**Random generation of CCP signatures**

For each cohort, we generated up to 10,000 signatures of sizes 1, 2, 4, 6, …30, 31 genes. Given a signature size *k*, we selected the lower of either *k* genes or all the genes profiled on the microarray. If <10,000 signatures of size *k* existed, then we randomly selected 10,000 signatures of size *k*. Otherwise, all possible gene signatures of size *k* were analyzed.

**Refinement of cell cycle proliferation signature**

Four refined CCP signatures were selected based on the prognostic value of the 31 CCP genes in BL and LA cohorts (**Figure S5**). Three signatures were selected using robust signature genes as follows: CCP-12 includes the 12 genes that were predictive of survival (P < 0.05) in ≥2 BL cohorts or ≥4 LA cohorts. CCP-10 includes the ten genes predictive of survival in 100% of BL cohorts or in at least 62.5% of LA cohorts where the gene was profiled. (We selected 62.5% because no gene was predictive in more than 5/8 = 62.5% of the LA cohorts). The genes in the CCP-9 signature include the *top hits* in each cohort (P < 0.05). The top hit in a specific cohort is defined as the most prognostic gene (having the lowest P-value) that is also significant (P < 0.05) in at least one additional cohort. CCP-4 contains a set of core genes that are common to the CCP-9, CCP-10, and CCP-12 signatures.

**Evaluation of refined cell cycle proliferation signatures**

Scores for the refined signatures were calculated according to the same algorithm used initially [[9](#_ENREF_9)], with qPCR data for the same set of 15 housekeeper (HK) genes and the appropriate subset of CCP genes. The rejection criteria for the final score for each sample were modified in two ways. First, the maximum acceptable number of failing genes was adjusted from 9 (out of 31) to 1/3 the number of genes in the signature. Second, the threshold of 0.5 for the standard deviation of the triplicate measurements of the CCP score was adjusted by a factor of sqrt(31/*n*), where *n* is the number of genes in the signature. Only two scores were rejected, both from the CCP-4 signature. Cox Proportional Hazards models were used to test the association of the optimized CCP signatures with time to biochemical recurrence, censored at 10 years. The test statistic was the partial likelihood ratio for the change in deviance from the full versus the reduced model, i.e. with and without the variable of interest. Effect size was measured by the hazard ratio (HR) per one unit of the CCP signature with a 95% confidence interval. Multivariate analyses included CAPRA-S, which is a summary of clinical risk that includes pre-operative PSA, pathologic Gleason score, surgical margins, extra-capsular extension, seminal vesicle invasion, and lymph node invasion [[22](#_ENREF_22)].

**Gene set enrichment and general statistical analyses**

The Database for Visualization and Annotated Discovery (DAVID, Ref #[[23](#_ENREF_23)]) was used for gene set enrichment analysis to identify overrepresented Gene Ontology (GO) terms and KEGG pathways in lists of genes. Default DAVID parameters were used for the analysis (i.e., a threshold count of two) and GO terms and KEGG pathways having a false discovery rate (FDR) below 20% were identified. Enrichment was with respect to the Affymetrix Human Genome U133A or U133 Plus 2.0 backgrounds in gene lists we developed (below) from cohorts profiled on these platforms; otherwise the Homo sapiens whole genome background was used. Gene lists were developed by identifying significantly predictive genes (P < 0.01) by the non-parametric Wilcoxon-rank sum test or the univariate cox proportional hazards models (logrank P-value), or were obtained from the literature (**Table S11**). With the exception of DAVID all statistical analyses were performed in *R* [[24](#_ENREF_24)].

The area under the receiver operating curve (AUC) was used to evaluate the ability of CCP score to discriminate between progressors and non-progressors, node positive (N+) and node negative (N0) patients, and patients with and without recurrent tumors. AUC was calculated such that a value > 0.50 corresponds to CCP score being higher in progressors than in non-progressors, higher in N+ patients than N0 patients, or higher in patients with recurrent tumors than in patients with non-recurrent tumors. Cox proportional hazards models were used to assess the prognostic value of the continuous signature score when clinical follow-up time was available. Kaplan-Meier curves were generated for patients with the top, middle, and bottom 33% of scores for visualization purposes and the HR and logrank P-value of the continuous score were reported.

Multivariate analyses were performed using logistic regression for qualitative responses (i.e., progression in the CNUH and Lindgren cohorts) and cox proportional hazards models for responses where follow-up time was available. In the multivariate analysis, a ‘final model’ was generated through forward step-wise addition of significant variables as assessed by a significant increase in log likelihoods (P < 0.05) according to the Chi-Square test statistic. *Best available* (BA) models were generated by developing *final models* as described above but by excluding CCP score from the analysis. If no variables were significant (i.e., as in the Blaveri cohort), then the single best variable was selected. The contribution of CCP score to each BA model was quantified by calculating the C-statistic [[25](#_ENREF_25)] and the Integrated Discrimination Improvement (IDI, Ref #[[26](#_ENREF_26)]). The C-statistic is the probability that among two randomly selected individuals, the individual with the higher risk score has a shorter time to event. The IDI can be interpreted as the increase in ‘average sensitivity’ minus the increase in ‘one minus specificity’ when CCP score is included. The *R* packages *Hmisc* and *survC1* were used to calculate C-statistics for qualitative responses and for censored time to event responses, respectively, and the *survIDINRI* [[27](#_ENREF_27)] package was used to calculate the IDI. Statistical significance was assessed by likelihood ratio test, which is equivalent to testing whether IDI > 0 [[28](#_ENREF_28)].

**SUPPORTING RESULTS AND DISCUSSION**

**Gene set enrichment analysis identifies cell cycle related genes as the most consistently prognostic class of biomarkers in bladder and lung adenocarcinoma**

Data from 1968 patients were examined for two outcomes (defined in **Materials and Methods**): tumor progression in patients with bladder cancer (BL) and survival in patients with BL, lung adenocarcinoma (LUAD), lung squamous cell carcinoma (LUSC), and head and neck squamous cell carcinoma (HNSCC). In each cohort we identified lists of genes predictive of outcome (P < 0.01) and performed a gene set enrichment analysis to identify overrepresented modules (GO terms and KEGG pathways, **Figure 2, Figure S1,** and **Table S5**). We also identified the *top hit* in each cohort, which we defined as the module with the lowest FDR that has FDR < 20% in multiple cohorts. In BL cohorts the most consistently enriched GO terms for survival were those related to the cell cycle, such as *cell cycle phase*, which were enriched in four (out of five) cohorts (FDR < 5%). *Cell cycle* was also the most consistently enriched KEGG pathway in three cohorts (FDR < 5%). In BL cohorts with progression as the endpoint, *cell cycle*, *cell cycle phase*, and *mitotic cell cycle* were the only three GO terms overrepresented in the predictive genes in all cohorts (FDR < 5%), while the only overrepresented KEGG pathway (FDR < 5%) was *cell cycle,* which was the top hit in each cohort. In patients with LUAD, the most consistently enriched GO terms (FDR < 5%) were those pertaining to cell cycle (e.g., *nuclear division*, *M phase of mitotic cell cycle*), and cell cycle related GO terms were top hits in five of eight LUAD cohorts. The most consistently enriched KEGG pathway was *cell cycle* in 4/8 cohorts. In the 3 cohorts that were not enriched in cell cycle related modules, top hits included *mRNA metabolic process*, *regulation of cell motion, and epidermis development*. In the 3 LUSC patient cohorts we found that that the GO term *negative regulation of transcription* was the top hit in two cohorts (FDR < 20%), and the KEGG pathway *Retinol metabolism* was also the top hit in two cohorts (FDR < 20%). There were no overrepresented GO terms or KEGG pathways in any HNSCC cohorts (FDR < 20%). In summary, the most consistently overrepresented modules were cell cycle related, and these were histology dependent.

**Predictive power of cell cycle expression signature (CCP score) depends on gene number**

The CCP signature consists of 31 highly correlated genes which are each presumably reporting on cell cycle proliferation. Because there is likely redundancy in the information provided by these correlated genes, we hypothesized that the performance of a signature with <31 genes would be comparable to the performance of the full 31 gene signature. Thus we set out to examine the relationship between CCP signature size and prognostic value. No single gene predicted survival (P < 0.05) in all BL and LUAD cohorts in which the full CCP signature was predictive, even when accounting for cohorts in which the gene was not profiled (**Figure S5**). To assess the effect of increasing signature size on prognostic value, we randomly selected up to 10,000 signatures of sizes 1-31 (see **Supporting Materials and Methods in File S1**) and tested whether these signatures predicted survival in the BL and LUAD cohorts where CCP score was predictive. With the exception of one cohort (MSKCC), all random signatures with 10-15 CCP genes were significantly predictive (P < 0.05) of survival in each cohort (**Figure S3**). In addition, although the P-values decreased with increasing signature size, the decrease leveled off once the signature size reached ~10 genes (**Figure S4**).

**Multivariate analysis**

We performed a multivariate analysis of CCP score along with clinically relevant variables such as age, gender, and grade in the BL and LA cohorts where CCP score was prognostic in the univariate analysis. For each cohort we also developed a best multivariate model (i.e., a *final model*) through forward stepwise selection of informative variables (P < 0.05). Such a model selects a concise, ‘optimal’ set of informative variables and may select CCP score over standard clinical variables. Because clinical variables are always readily available, such models may not be cost-effective currently but are expected to be so in settings where cancers are staged and graded according to their molecular pathology.

We first assessed the clinical utility of CCP score to predict progression in patients with BL. CCP score was the only significantly predictive (P < 0.05) variable in the multivariate analysis (Dyrskjot cohort) and the only variable selected for inclusion in the *final model* in all three cohorts (**Table 1 and Table S6 in File S4**). Therefore, CCP score was the strongest prognostic variable for progression in these datasets. We next evaluated CCP score in BL patients in a multivariate survival analysis (DSS or OS) using clinical variables that would be available in a preoperative setting (**Table 1 and Table S7 in File S4**). Higher CCP scores were strongly associated with increased risk (P < 0.05) in three datasets (Blaveri, Dyrskjot, MSKCC) in the multivariate analysis. Patients with MI tumors also had an increased risk (P < 0.05) in three cohorts (CNUH, Lindgren, MSKCC). No other variables were significant in multiple cohorts in multivariate analysis. CCP score and stage were also consistently included in the *final model* and were selected 2 and 3 times, respectively. Age was also included in the *final model* in two cohorts

In patients with LA, clinical variables such as stage, grade, chemotherapy treatment, and smoking history were available in four of the five cohorts where CCP score was prognostic, allowing for a multivariate survival analysis (OS or RFS) (**Table 2 and** **Table S8 in File S4**). CCP score was the most consistently predictive variable in the multivariate analysis, with significance (P < 0.05) in three cohorts while no other variable was significant in more than one. Furthermore, CCP score was the variable most frequently included in the *final models* (in three cohorts), followed by stage (in two cohorts).

We next evaluated whether CCP score increases the prognostic power of the *best available* (BA) models that include readily available clinical variables, as described in **Supporting Materials and Methods in File S1.** The addition of CCP score to the BA model led to a significant improvement in two (out of three) BL cohorts when progression was the endpoint, in one (out of five) BL cohorts when survival was the endpoint, and three (out of four) LUAD cohorts when survival was the endpoint (**Tables S9-S10 in File S5**). Altogether, these results indicate that CCP score is a robust prognostic variable for progression in BL patients and for survival in BL and LA patients and that CCP score can augment multivariate clinical risk models of survival for these cancers. Specifically, our results suggest that CCP score may have immediate clinical utility as a prognostic marker for progression in BL patients and survival in LUAD patients, and that prospective evaluation of CCP score is warranted in these cancers.

**Published bladder cancer and lung adenocarcinoma signatures**

We analyzed 4 BL progression signatures and 10 survival signatures that predict outcome in patients with LUAD (**Table S11**). Several LUAD survival signatures were developed from the UM and HLM cohorts (**Table S2 in File S2**) using various methods in a multi-site, blinded validation study [[17](#_ENREF_17)]. This work evaluated several gene signatures and we analyzed all available signatures which were found to be predictive in their study. These signatures included Methods C,D,F, and G (25-37 genes) and Method H (250 genes). For the discussion regarding the specific signature genes see **Supporting Materials and Methods in File S1**.

We identified commonalities in BL progression and LA survival signatures (**Table S11**) at the gene level and functional level by gene set enrichment analysis. The BL progression signatures contain four genes that are common to two or more signatures, with a median overlap of 4% of genes per signature. The common genes include GSTM4 (Wang and Lindgren signatures) and SF3B1, CHUK and SMARCA4 (Dyrskjot and Lindgren signatures). The latter two genes are involved in regulation of DNA binding transcription factor activity, according to the GO database [[29](#_ENREF_29)]. The Dyrskjot signature is enriched in cell cycle related (e.g., *M phase*) GO terms, as well as the *cell cycle* KEGG pathway (**Table S13**). However, there were no enriched GO terms or KEGG pathways common to multiple progression signatures at FDR < 20%.

The LA progression signatures included two genes that are common to four signatures. ABAT is a metabolic gene in the Method C, D, F, and G signatures; however these signatures were all derived from the HLM and UM cohorts. MCM6 is a cell cycle related gene that was independently identified in the Bianchi, Kadara, and Method H signatures. The Method C, D, F, and G signatures had an average overlap of 28% of genes per signature. All five Bianchi signature genes were part of the Method H signature. Not counting the Bianchi signature, there was a mean overlap of 11% of signature genes across all signatures when the Method C, D, F, and G signatures were combined. The only GO terms overrepresented (FDR < 0.20) in multiple signatures were cell-cycle related (e.g., *DNA-dependent DNA replication*), which were overrepresented in two signatures (Kadara, MethodH) at a FDR < 1% (**Table S13**). In addition, the top hit for MethodD was *positive regulation of apoptosis* (FDR < 20%). Three signatures (Bianchi, Kadara, MethodH) were enriched in cell cycle-related KEGG pathways (*DNA replication*, Kadara and MethodH, FDR < 1%; *cell cycle*, Bianchi and MethodH, FDR < 10%). No other GO terms or KEGG pathways were overrepresented in multiple signatures at an FDR < 20%.

The analysis of these signatures and their adjustment for CCP score (**Figure 4**) characterizes the importance of cell cycle in prognostic biomarker signatures in two ways. First, the only consistently enriched modules in the gene signatures were cell cycle related. However, this common enrichment was rare and limited to LA signatures (Bianchi, Kadara, and Method H signatures). Secondly, both BL progression and LA survival signatures lost their predictive ability regardless of whether or not they were associated with cell cycle. Specifically, although only the Dyrskjot BL progression signature was enriched in cell cycle related modules, the Wang and CNUH signatures lost their predictive ability in 100% of the BL cohorts that they were prognostic in. The Lindgren signature lost its predictive ability in all independent datasets it was tested in. Among the LA signatures, the Method F and Method G signatures were not enriched in cell cycle related modules but lost their predictive ability in 71% and 80% of cohorts, respectively. In fact, all LA signatures except for Tomida lost their predictive ability in ≥50% of the cohorts. Importantly, a loss in predictive ability indicates that signature genes were correlated with CCP score. This implies that many signatures were enriched in genes that correlate with cell cycle activity, even though the signature genes were generally not annotated as such.

**Prognostic signatures in bladder and lung carcinoma depend on cell cycle related genes**

In each dataset, expression levels of signature genes were adjusted for CCP score or by a constant term comprising a “negative control” (see **Supporting Materials and Methods in File S1** for details) and evaluated for progression in the BL cohorts and survival in the LUAD cohorts. A heatmap indicates each signature’s prognostic value and whether its predictive ability is lost when adjusted for CCP score (**Figure 4**). Each BL progression signature was prognostic in two cohorts (P < 0.05, **Figure 4A**). However, the Wang, Dyrskjot, and CNUH signatures completely lost their predictive ability following CCP adjustment. The Lindgren signature lost its predictive ability in the CNUH but not the Lindgren cohort, possibly because of overfitting (signature derived and evaluated in the same cohort). The LUAD outcome signatures lost their predictive ability in 71% of the cohorts when adjusted for CCP score (**Figure 4B**). The MethodD and MethodH signatures were the most robust signatures and were both predictive in 7/8 cohorts but lost predictive ability in 85% and 71% of the cohorts, respectively, when adjusted by CCP-score. The Kadara and Bianchi signatures were both prognostic in 4 cohorts and lost their predictive ability in all. Only one LUAD signature (Tomida) maintained its predictive ability following CCP-adjustment but was prognostic (P < 0.05) in only two cohorts.

**REFERENCES**

1. Barrett T, Troup DB, Wilhite SE, Ledoux P, Evangelista C, et al. (2011) NCBI GEO: archive for functional genomics data sets--10 years on. Nucleic acids research 39: D1005-1010.

2. Parkinson H, Sarkans U, Kolesnikov N, Abeygunawardena N, Burdett T, et al. (2011) ArrayExpress update-an archive of microarray and high-throughput sequencing-based functional genomics experiments. Nucleic Acids Research 39: D1002-D1004.

3. Blaveri E, Simko JP, Korkola JE, Brewer JL, Baehner F, et al. (2005) Bladder cancer outcome and subtype classification by gene expression. Clin Cancer Res 11: 4044-4055.

4. Sanchez-Carbayo M, Socci ND, Lozano J, Saint F, Cordon-Cardo C (2006) Defining molecular profiles of poor outcome in patients with invasive bladder cancer using oligonucleotide microarrays. J Clin Oncol 24: 778-789.

5. Chung CH, Parker JS, Ely K, Carter J, Yi Y, et al. (2006) Gene expression profiles identify epithelial-to-mesenchymal transition and activation of nuclear factor-kappaB signaling as characteristics of a high-risk head and neck squamous cell carcinoma. Cancer Research 66: 8210-8218.

6. Shimizu S, Tsukada J, Sugimoto T, Kikkawa N, Sasaki K, et al. (2008) Identification of a novel therapeutic target for head and neck squamous cell carcinomas: a role for the neurotensin-neurotensin receptor 1 oncogenic signaling pathway. Int J Cancer 123: 1816-1823.

7. Colombo J, Fachel AA, De Freitas Calmon M, Cury PM, Fukuyama EE, et al. (2009) Gene expression profiling reveals molecular marker candidates of laryngeal squamous cell carcinoma. Oncol Rep 21: 649-663.

8. Hastie T, Ribshirani R, Balasubramanian N, Chu G (2010) impute: Imputation for microarray data.

9. Cuzick J, Swanson GP, Fisher G, Brothman AR, Berney DM, et al. (2011) Prognostic value of an RNA expression signature derived from cell cycle proliferation genes in patients with prostate cancer: a retrospective study. Lancet Oncol 12: 245-255.

10. Dyrskjot L, Zieger K, Real FX, Malats N, Carrato A, et al. (2007) Gene expression signatures predict outcome in non-muscle-invasive bladder carcinoma: a multicenter validation study. Clin Cancer Res 13: 3545-3551.

11. Lee ES, Son DS, Kim SH, Lee J, Jo J, et al. (2008) Prediction of Recurrence-Free Survival in Postoperative Non-Small Cell Lung Cancer Patients by Using an Integrated Model of Clinical Information and Gene Expression. Clinical Cancer Research 14: 7397-7404.

12. Lindgren D, Frigyesi A, Gudjonsson S, Sjodahl G, Hallden C, et al. (2010) Combined gene expression and genomic profiling define two intrinsic molecular subtypes of urothelial carcinoma and gene signatures for molecular grading and outcome. Cancer Res 70: 3463-3472.

13. Wang R, Morris DS, Tomlins SA, Lonigro RJ, Tsodikov A, et al. (2009) Development of a Multiplex Quantitative PCR Signature to Predict Progression in Non-Muscle-Invasive Bladder Cancer. Cancer Research 69: 3810-3818.

14. Bianchi F, Nuciforo P, Vecchi M, Bernard L, Tizzoni L, et al. (2007) Survival prediction of stage I lung adenocarcinomas by expression of 10 genes. J Clin Invest 117: 3436-3444.

15. Kadara H, Behrens C, Yuan P, Solis L, Liu D, et al. (2011) A five-gene and corresponding protein signature for stage-I lung adenocarcinoma prognosis. Clin Cancer Res 17: 1490-1501.

16. Onaitis M, D'Amico TA, Clark CP, Guinney J, Harpole DH, et al. (2011) A 10-gene progenitor cell signature predicts poor prognosis in lung adenocarcinoma. Annals of Thoracic Surgery 91: 1046-1050; discussion 1050.

17. Shedden K, Taylor JMG, Enkemann SA, Tsao MS, Yeatman TJ, et al. (2008) Gene expression-based survival prediction in lung adenocarcinoma: a multi-site, blinded validation study. Nature Medicine 14: 822-827.

18. Tomida S, Takeuchi T, Shimada Y, Arima C, Matsuo K, et al. (2009) Relapse-related molecular signature in lung adenocarcinomas identifies patients with dismal prognosis. J Clin Oncol 27: 2793-2799.

19. Eyre TA, Ducluzeau F, Sneddon TP, Povey S, Bruford EA, et al. (2006) The HUGO Gene Nomenclature Database, 2006 updates. Nucleic Acids Res 34: D319-321.

20. Mosley JD, Keri RA (2008) Cell cycle correlated genes dictate the prognostic power of breast cancer gene lists. BMC Med Genomics 1: 11.

21. Tibshirani R, Hastie T, Narasimhan B, Chu G (2002) Diagnosis of multiple cancer types by shrunken centroids of gene expression. Proc Natl Acad Sci U S A 99: 6567-6572.

22. Cooperberg MR, Hilton JF, Carroll PR (2011) The CAPRA-S Score A Straightforward Tool for Improved Prediction of Outcomes After Radical Prostatectomy. Cancer 117: 5039-5046.

23. Huang da W, Sherman BT, Lempicki RA (2009) Systematic and integrative analysis of large gene lists using DAVID bioinformatics resources. Nat Protoc 4: 44-57.

24. R: A Language and Environment for Statistical Computing. Vienna (AU): R Development Core Team.

25. Uno H, Cai T, Pencina MJ, D'Agostino RB, Wei LJ (2011) On the C-statistics for evaluating overall adequacy of risk prediction procedures with censored survival data. Stat Med 30: 1105-1117.

26. Pencina MJ, D'Agostino RB, Sr., Steyerberg EW (2011) Extensions of net reclassification improvement calculations to measure usefulness of new biomarkers. Stat Med 30: 11-21.

27. Uno H, Tian L, Cai T, Kohane IS, Wei LJ (2012) A unified inference procedure for a class of measures to assess improvement in risk prediction systems with survival data. Stat Med.

28. Pepe MS, Feng Z, Gu JW (2008) Comments on 'Evaluating the added predictive ability of a new marker: From area under the ROC curve to reclassification and beyond' by M. J. Pencina et al., Statistics in Medicine (DOI: 10.1002/sim.2929). Stat Med 27: 173-181.

29. Ashburner M, Ball CA, Blake JA, Botstein D, Butler H, et al. (2000) Gene ontology: tool for the unification of biology. The Gene Ontology Consortium. Nat Genet 25: 25-29.
