# Supplementary material for: Robust Prognostic Gene Expression Signatures in Bladder Cancer and Lung Adenocarcinoma Depend on Cell Cycle Related Genes
Source: PLoS One. 2014 Jan 22;9(1):e85249. doi: 10.1371/journal.pone.0085249 (PMC3898982; doi:10.1371/journal.pone.0085249)
Supplement: File S2 — Summary of bladder cancer (Table S1), lung adenocarcinoma (Table S2), lung squamous cell carcinoma (Table S3), and head and neck squamous cell carcinoma (Table S4) patient cohorts. (PDF) [file pone.0085249.s002.pdf]

Supplementary Table S1. Bladder cancer patient cohorts

				Stage		Grade				
**Cohort***	**Platform**	**# CCP genes**	**N**	**NMI**	**MI**	**LG**	**HG**	**Progression**	**Endpoint (survival)**	**Ref**
**Blaveri**	UCSF Human array 2.0	16	74	27	47	10	62	-	OS	(1)
**CNUH (GSE13507)**	Illumina human-6 v2.0	30	165	104	61	105	60	Any increase in stage	DSS	(2)
**Dyrskjot (GSE5479)**	MDL human 3k oligo array	5	366	351	45	130	267	NMI → MI	PFS; DSS	(3)
**Lindgren (GSE11915)**	Swegene	11	142	97	43	72	70	NMI → MI	DSS	(4)
**MSKCC**	HG-U133A	31	87	22	65	17	70	-	OS	(5)

*GSE cohorts are available from Gene Expression Omnibus (6)
Abbreviations: NMI, non-muscle invasive (Ta-T1); MI, muscle invasive (T2-T4); LG, low grade (G1-G2); HG, high grade (G3-G4); OS, overall survival; DSS, disease specific survival; PFS, progression-free survival

Supplementary Table S2. Lung adenocarcinoma patient cohorts

				Stage			Grade (differentiation)			Smoking history			
**Cohort***	**Platform**	**# CCP genes**	**N**	**I**	**II**	**III**	**Low**	**Moderately**	**Well**	**Never**	**Current or former**	**Endpoint (survival)**	**Ref**
**Bild (GSE3141)**	HG U133 Plus 2.0	31	58	-			-			-		OS	(7)
**Son (GSE8894)**	HG U133 Plus 2.0	31	62	-			-			-		RFS	(8)
**Takeuchi (GSE11969)**	Agilent custom microarray	26	90	52	13	25	25	31	34	45	45	OS	(9)
**Tomida (GSE13213)**	Agilent -014850	31	117	79	13	25	-			56	61	OS	(10)
**MSK**	HG U133A	31	104	63	20	21	13	41	46	19	83	OS	(11)
**CAN/DF**	HG U133A	31	82	56	26	0	37	31	13	10	68	OS	(11)
**UM**	HG U133A	31	178	116	29	33	66	86	26	11	79	OS	(11)
**HLM**	HG U133A	31	79	41	20	15	18	51	8	9	70	OS	(11)

*GSE cohorts are available from Gene Expression Omnibus (6)
Abbreviations: OS, overall survival; RFS, recurrence-free survival

Supplementary Table S3. Lung squamous cell carcinoma patient cohorts

				Stage			Grade (differentiation)			Smoking history			
**Cohort***	**Platform**	**# CCP genes**	**N**	**I**	**II**	**III**	**Low**	**Moderately**	**Well**	**Never**	**Current or former**	**Endpoint (survival)**	**Ref**
**Bild (GSE3141)**	HG U133 Plus 2.0	31	53		-			-		-		OS	(7)
**Son (GSE8894)**	HG U133 Plus 2.0	31	76		-			-		-		RFS	(8)
**Takeuchi (GSE11969)**	Agilent custom microarray	26	35	17	5	13		-		1	34	OS	(9)

*GSE cohorts are available from Gene Expression Omnibus (6)
Abbreviations: OS, overall survival; RFS, recurrence-free survival

Supplementary Table S4. Head and neck squamous cell carcinoma patient cohorts

Cohort*	Platform	#CCP genes	N	Nodal involvement (N0,N+)	Endpoint (survival)	Ref
**Cohen (GSE10300)**	HG U133 Plus 2.0	31	44	-	RFS	(12)
**Colo (GSE18020)**	FAP Human 4.8K03	5	81	(20,61)	Recurrence	(13)
**Colombo (GSE10288)**	CAGE lab custom microarray	0	12	-	RFS	
**Pavon (GSE23036)**	HG U133 Plus 2.0	31	63	(10,53)	-	(6)

*GSE cohorts are available from Gene Expression Omnibus (6)
Abbreviations: RFS, recurrence-free survival

REFERENCES

1. Blaveri E, Simko JP, Korkola JE, et al. Bladder cancer outcome and subtype classification by gene expression. Clin Cancer Res. 2005;11(11):4044-55. Epub 2005/06/03.
2. Kim WJ, Kim EJ, Kim SK, et al. Predictive value of progression-related gene classifier in primary non-muscle invasive bladder cancer. Molecular Cancer. 2010;9:3. Epub 2010/01/12.
3. Dyrskjot L, Zieger K, Real FX, et al. Gene expression signatures predict outcome in non-muscle-invasive bladder carcinoma: a multicenter validation study. Clin Cancer Res. 2007;13(12):3545-51. Epub 2007/06/19.
4. Lindgren D, Frigyesi A, Gudjonsson S, et al. Combined gene expression and genomic profiling define two intrinsic molecular subtypes of urothelial carcinoma and gene signatures for molecular grading and outcome. Cancer Res. 2010;70(9):3463-72. Epub 2010/04/22.
5. Sanchez-Carbayo M, Socci ND, Lozano J, Saint F, Cordon-Cardo C. Defining molecular profiles of poor outcome in patients with invasive bladder cancer using oligonucleotide microarrays. J Clin Oncol. 2006;24(5):778-89. Epub 2006/01/25.
6. Barrett T, Troup DB, Wilhite SE, et al. NCBI GEO: archive for functional genomics data sets-10 years on. Nucleic Acids Research. 2011;39:D1005-D10.
7. Bild AH, Yao G, Chang JT, et al. Oncogenic pathway signatures in human cancers as a guide to targeted therapies. Nature. 2006;439(7074):353-7.
8. Lee ES, Son DS, Kim SH, et al. Prediction of Recurrence-Free Survival in Postoperative Non-Small Cell Lung Cancer Patients by Using an Integrated Model of Clinical Information and Gene Expression. Clinical Cancer Research. 2008;14(22):7397-404.
9. Takeuchi T, Tomida S, Yatabe Y, et al. Expression profile-defined classification of lung adenocarcinoma shows close relationship with underlying major genetic changes and clinicopathologic behaviors. Journal of Clinical Oncology. 2006;24(11):1679-88.
10. Tomida S, Takeuchi T, Shimada Y, et al. Relapse-related molecular signature in lung adenocarcinomas identifies patients with dismal prognosis. J Clin Oncol. 2009;27(17):2793-9. Epub 2009/05/06.
11. Shedden K, Taylor JMG, Enkemann SA, et al. Gene expression-based survival prediction in lung adenocarcinoma: a multi-site, blinded validation study. Nature Medicine. 2008;14(8):822-7.
12. Cohen EE, Zhu H, Lingen MW, et al. A feed-forward loop involving protein kinase Calpha and microRNAs regulates tumor cell cycle. Cancer Research. 2009;69(1):65-74. Epub 2009/01/02.
13. Colo AE, Simoes AC, Carvalho AL, et al. Functional microarray analysis suggests repressed cell-cell signaling and cell survival-related modules inhibit progression of head and neck squamous cell carcinoma. BMC Med Genomics. 2011;4:33. Epub 2011/04/15.
