# Supplementary material for: Robust Prognostic Gene Expression Signatures in Bladder Cancer and Lung Adenocarcinoma Depend on Cell Cycle Related Genes
Source: PLoS One. 2014 Jan 22;9(1):e85249. doi: 10.1371/journal.pone.0085249 (PMC3898982; doi:10.1371/journal.pone.0085249)
Supplement: File S3 — Sweave document containing sample R code and output. (PDF) [file pone.0085249.s003.pdf]

Supporting Information S3

Sample R Code for 'Robust prognostic gene expression signatures in bladder and lung adenocarcinoma depend on cell cycle related genes'

Contents

1 Univariate analysis of CCP score in CNUH 1

2 *R* functions for multivariate analysis and development of the *final* progression and survival models 2

3 Development of the *final model* for predicting progression in CNUH 8

4 *R* functions for assessing the improvement in prognostic power when CCP score is added to the *best available* model 11

5 Assessing the improvement in prognostic power when CCP score is added to the *best available* progression and survival models for CNUH 18

6 *R* function for adjusting gene expression data by a specified score (e.g., CCP) 20

1 Univariate analysis of CCP score in CNUH

```
> ##############################################################################
> ### This code evaluates the CCP signature in the CNUH (Korea, GSE13507) cohort
> ##############################################################################
> GENE.LIST = read.table("CCP.genes.txt")
> ## load the CNUH (Korea, GEO Acc# 13507) dataset with only CCP genes
```

```
> ## (set by GENE.LIST above) ##
> loadKorea()

loading Korea data...
loading gene expression data...
loading platform (GPL6102)...
1 matching probes for gene symbol FOXM1
1 matching probes for gene symbol CDC20
1 matching probes for gene symbol CDKN3
3 matching probes for gene symbol CDC2 /// CDK1
1 matching probes for gene symbol KIF11
2 matching probes for gene symbol KIAA0101
1 matching probes for gene symbol NUSAP1
1 matching probes for gene symbol CENPF
1 matching probes for gene symbol ASPM
1 matching probes for gene symbol BUB1B
1 matching probes for gene symbol RRM2
No matching probes for gene symbol DLGAP5
1 matching probes for gene symbol BIRC5
1 matching probes for gene symbol KIF20A
1 matching probes for gene symbol PLK1
1 matching probes for gene symbol TOP2A
1 matching probes for gene symbol TK1
1 matching probes for gene symbol PBK
1 matching probes for gene symbol ASF1B
1 matching probes for gene symbol C18orf24 /// SKA1
3 matching probes for gene symbol RAD54L
1 matching probes for gene symbol PTTG1
1 matching probes for gene symbol CDCA3
1 matching probes for gene symbol MCM10
1 matching probes for gene symbol PRC1
1 matching probes for gene symbol DTL
1 matching probes for gene symbol CEP55
2 matching probes for gene symbol RAD51
2 matching probes for gene symbol CENPM
1 matching probes for gene symbol CDCA8
1 matching probes for gene symbol ORC6L /// ORC6

> ## ccp score is average of normalized genes ##
> korea.score = apply(Z.normalize(GSE13507.expr, 1),2,mean)
```

```
> col = c("green", "blue", "red")
> probs = c(1/3, 2/3)
> lwd = 5
> plot.km.paper(GSE13507.DSS.time, GSE13507.DSS.outcome,
+               factor(bin(korea.score,probs)), col = col, line.style = 1,
+               h.str="", p.str = "", NO.LEGEND=T, max.x = 60, line.width = lwd,
+               ylab = "DSS (%)", xlab = "time (months)")
```

```
sorting legend in decreasing order for values, line.style, col...
```

```
> coxph(Surv(GSE13507.DSS.time, GSE13507.DSS.outcome) ~ korea.score)
```

```
Call:
coxph(formula = Surv(GSE13507.DSS.time, GSE13507.DSS.outcome) ~
    korea.score)


             coef exp(coef) se(coef)    z       p
korea.score 0.865      2.38    0.222 3.91 9.4e-05

Likelihood ratio test=16.6  on 1 df, p=4.74e-05  n= 165, number of events= 32
   (90 observations deleted due to missingness)
```

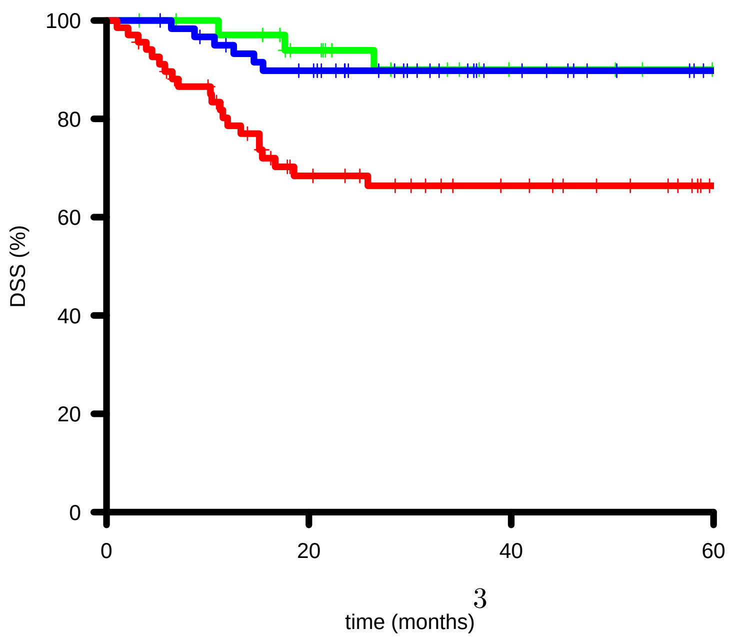

Figure 1: Prognostic value of CCP score for survival in CNUH cohort, plotted for high (red), intermediate (blue) and low (green) values of CCP score

2 *R* functions for multivariate analysis and development of the *final* progression and survival models

```
> ############################################################################
> ## Multivariate Regression Functions                                      ##
> ############################################################################
>
> library(survival)
> library(inference)
> ##############################################################################
> # Performs univariate, multivariate, and stepwise analysis for logistic     #
> # regression or cox proportional hazards models
> #        - X: data matrix (with columns as variables)                        #
> #        - Y: response variable, either a vector of responses or a list      #
> #                with components time and outcome                            #
> #        - mod: the full model, e.g., ~ a + b + c + d                        #
> #        - func: either 'logistic' or 'coxph'; if NULL, then coxph is chosen #
> #                if 'Y' is a list; otherwise 'logistic' will be used         #
> #        - subset: rows of 'X' to use; if 'NULL', then removes all rows      #
> #                NA
> # Details: fits model to individual terms, all terms, and performs          #
> #        forward regression, keeping variables with added value and          #
> #        P < 0.05 based on chi-square test                                   #
> # Note: This will update the variable data.X in the global environment      #
> ##############################################################################
> multivariate.regression <- function(X,Y, mod, func = NULL, p.cut = 0.05,
+         subset = rep(TRUE, nrow(X)), scale.est = TRUE, robust.se = TRUE) {
+   if (is.null(mod)) stop("ERROR: MUST ENTER mod!\n")
+   if (is.null(subset)) subset = apply(is.na(X), 1, sum)==0
+
+   X = X[subset,]
+   if (is.list(Y)) {
+         Y$time = Y$time[subset]; Y$outcome = Y$outcome[subset]
+         cat("multivariate analysis with n = ",
+                 sum(!is.na(Y$time) & !is.na(Y$outcome)), "\n")
+   } else {
+         Y = Y[subset]
+         cat("multivariate analysis with n = ", sum(!is.na(Y)), "\n")
```

```
+   }
+
+   data.X <<- X
+   ##############################################################
+   ### check input arguments                                   ##
+   ##############################################################
+   if (is.null(func)) {
+         if (is.list(Y)) {
+                 func = "coxph"
+         } else {
+                 func = "logistic"
+         }
+   }
+   if (!func %in% c("logistic", "coxph")) {
+     stop("func must be logistic or coxph...\n")
+   }
+    n1 = nrow(data.X)
+    n2 = length(Y)
+    if (func == "coxph") n2 = length(Y$time)
+    if (n1!=n2) {
+     stop("variables should correspond to columns of data.X\n")
+    }
+
+   ##############################################################
+   ### Univariate analysis                                     ##
+   ##############################################################
+    ans.ind = NULL
+    for (i in 1:ncol(data.X)) {
+      if (func == "logistic") {
+        g.ind = glm(Y ~ data.X[,i], family = "binomial")
+        add.this = infer.format(g.ind, scale.est, robust.se)
+        if (nrow(add.this) == 1) ans.ind = rbind(ans.ind, NA)
+        if (nrow(add.this) > 1)  ans.ind = rbind(ans.ind, add.this[2,])
+      } else if (func == "coxph"){
+        g.ind = coxph(Surv(Y$time, Y$outcome) ~ data.X[,i])
+        add.this = infer.format(g.ind, scale.est, robust.se)
+        ans.ind = rbind(ans.ind, add.this)
+      }
+    }
+    rownames(ans.ind) = colnames(data.X)
```

```
+    colnames(ans.ind) = c("Univariate.Estimate", "95% CI", "Univariate.P-value")
+    if (scale.est & func == "coxph") colnames(ans.ind)[1] = "Univariate.HR"
+    if (scale.est & func == "logistic") colnames(ans.ind)[1] = "Univariate.Odds.Rati
+
+   ##############################################################
+   ### Multivariate analysis                                  ##
+   ##############################################################
+    if (func == "logistic") {
+      g.full = glm(Y ~ ., data = data.X, family = "binomial")
+      coef = coefficients(g.full)
+      g.full = infer.format(g.full, scale.est, robust.se)
+      g.full = g.full[-1,]  ## remove intercept
+    } else if (func == "coxph") {
+              g.full = coxph(Surv(Y$time, Y$outcome) ~ ., data = data.X)
+         g.full = infer.format(g.full, scale.est, robust.se)
+    }
+    g = NULL
+    if (func == "logistic") {
+         g = glm(Y ~ 1, data = data.X, family = "binomial")
+    } else if (func == "coxph") {
+         g = coxph(Surv(Y$time, Y$outcome) ~ 1, data = data.X)
+    }
+   ##############################################################
+   ### Forward stepwise regression                            ##
+   ##############################################################
+    f.m = step(g, scope = mod, direction = "forward")
+    cat("done...\n")
+    #return(list(g = g, fm = f.m))
+    #### which variables do we keep ####
+    a = anova(f.m, test = "Chisq")
+    ##return(list(a = a, g= g, fm =f.m))
+    i = ncol(a)
+    keep= a[,i] <= p.cut
+    keep[is.na(keep)] = FALSE
+    if (sum(keep) == 0) {
+         keep = NULL
+    } else {
+      keep = (rownames(a)[keep])
+    }
+    ######################################
```

```
+
+    nothing = matrix(" ", ncol = 3, nrow = nrow(g.full))
+    g = NULL
+    ## find final model ##
+    if (!is.null(keep)) {
+       m = match(keep, colnames(data.X))
+       final.data = data.frame(data.X[,m])
+     final.data = data.frame(data.X[,m])
+     colnames(final.data) = colnames(data.X)[m]
+     cat("getting final model...\n")
+       if (func == "logistic") {
+         g = glm(Y ~ ., data = final.data, family = "binomial")
+       } else if (func == "coxph") {
+         g = coxph(Surv(Y$time, Y$outcome) ~ ., data = final.data)
+       }
+       ##return(g)
+       g = infer.format(g, scale.est, robust.se)
+       if (func == "logistic") {
+         nn = rownames(g); g = g[-1,]
+         if (is.null(nrow(g))){
+                 g = t(g); rownames(g) = nn[-1]
+         }
+       }
+       m = match(rownames(g), rownames(g.full))
+       m = m[!is.na(m)]
+       if (length(m) > 0) nothing[m,] = g
+       colnames(nothing) = colnames(g)
+    }
+    ans = data.frame(cbind(g.full, nothing))
+    colnames(ans) = c("Full.Estimate", "95% CI", "Full.P-value",
+                      "Final.Estimate", "95% CI", "Final.P-value")
+   if (scale.est & func == "coxph")  {
+     colnames(ans)[1] = "Full.HR"; colnames(ans)[4] = "Final.HR"
+   } else if (scale.est & func == "logistic") {
+     colnames(ans)[1] = "Full.Odds.Ratio"
+     colnames(ans)[4] = "Final.Odds.Ratio"
+   }
+    ans = cbind(ans.ind, ans)
+    return(ans)
+ }
```

```
> ##########################################################################
> ## inference function: returns estimate, 95% CI and P-value from 'g.mod' #
> ## scale.est: if TRUE, scales exponentially scales estimates which       #
> ##    results in HR for coxph model and Odds Ratio for logistic model    #
> ##########################################################################
> infer.format <- function(g.mod, scale.est = TRUE, robust.se = FALSE) {
+   est = infer(g.mod, robust.se = robust.se)
+   conf = cbind(est[,4], est[,5])
+   p.values = est[,3]
+   est = est[,1]
+   if (scale.est) {est = exp(est); conf = exp(conf)}
+   tmp = apply(round(conf,2), 1,paste, collapse = ", ")
+   conf =  paste("(",tmp, ")", sep = "")
+   ans = cbind(Estimate = est, "95% CI" = conf, p.value = p.values)
+   if (nrow(ans) == 1) rownames(ans) =  names(g.mod$coefficients)[1]
+    if (scale.est) {
+         if("glm" %in%attr(g.mod, "class")) colnames(ans)[1] = "Odds.Ratio"
+         if("coxph" %in%attr(g.mod, "class")) colnames(ans)[1] = "HR"
+    }
+    return(ans)
+ }
> format.multivariate <-function(x) {
+   cols = (1:ncol(x))[colnames(x)!= "95% CI"]
+   round.it <-function(x,d = 3) round(to.d(x),d)
+   to.d <- function(x) as.double(as.character(x))
+
+   for (i in cols) {
+       x[,i] = round.it(x[,i])
+   }
+   x[is.na(x)] = ""
+   return(x)
+ }
>
```

3 Development of the *final model* for predicting progression in CNUH

```
> ################################################################
> ## multivariate analysis of progression in CNUH
```

```
> ####################################################################
> is.value <- function(x,val) {
+         index = x == val
+         index[is.na(index)] = FALSE
+         return(index)
+ }
> #no.na <- function(X) { return(apply(!apply(X,1,is.na), 2, all)) }
>
> korea.progression = rep(NA, nrow(GSE13507.p))
> g1 = grep("progression", GSE13507.p$characteristics_ch1.7)
> g2 = grep("progression", GSE13507.p$characteristics_ch1.9)
> a = as.character(GSE13507.p$characteristics_ch1.7[g1])
> b = as.character(GSE13507.p$characteristics_ch1.9[g2])
> korea.progression[g1] = a
> korea.progression[g2] = b
> tmp = rep(NA, length(korea.progression))
> tmp[korea.progression == "progression: Yes"] = 2
> tmp[korea.progression == "progression: No"] = 1
> korea.progression = tmp
> data.korea = data.frame(CCP = korea.score, stage = Korea.stage,
+                         grade = Korea.grade,
+                         bcg = as.integer(is.value(GSE13507.intravesical,1)),
+                         chemo = as.integer(is.value(GSE13507.chemo,1)),
+                         age = GSE13507.age, gender = GSE13507.gender)
> korea.mod = ~ CCP + stage + grade + bcg + chemo + age + gender
> mm.korea.p =
+     multivariate.regression(data.korea, korea.progression-1, korea.mod)

multivariate analysis with n =  165
Start:  AIC=161.43
Y ~ 1

        Df Deviance    AIC
+ stage   1   147.40 151.40
+ grade   1   149.55 153.55
+ CCP     1   149.89 153.89
+ chemo   1   153.30 157.30
<none>        159.43 161.43
+ age     1   157.77 161.77
+ bcg     1   158.27 162.27
```

```
+ gender  1   159.40 163.40

Step:  AIC=151.4
Y ~ stage

         Df Deviance    AIC
+ CCP     1   143.01 149.01
+ grade   1   144.86 150.86
<none>        147.40 151.40
+ bcg     1   145.56 151.56
+ age     1   146.45 152.45
+ chemo   1   146.85 152.85
+ gender  1   147.40 153.40

Step:  AIC=149.01
Y ~ stage + CCP

         Df Deviance    AIC
<none>        143.01 149.01
+ bcg     1   141.48 149.48
+ grade   1   142.21 150.21
+ chemo   1   142.29 150.29
+ age     1   142.77 150.77
+ gender  1   142.79 150.79
done...
getting final model...

> format.multivariate(mm.korea.p)

       Univariate.Odds.Ratio       95% CI
CCP                    2.077  (1.29, 3.38)
stage                  4.124 (1.81, 9.39)
grade                  3.591  (1.6, 8.08)
bcg                    0.623  (0.26, 1.5)
chemo                  3.277 (1.32, 8.13)
age                    1.023 (0.99, 1.06)
gender                 0.909 (0.34, 2.46)
       Univariate.P-value Full.Odds.Ratio        95% CI
CCP                 0.003           1.524  (0.84, 2.76)
stage               0.001           3.584 (0.84, 15.29)
```

```
grade                0.002           1.631  (0.60, 4.44)
bcg                  0.291           2.469 (0.61, 10.05)
chemo                0.010           1.927  (0.59, 6.23)
age                  0.173           1.016  (0.97, 1.06)
gender               0.851           1.329  (0.47, 3.68)
       Full.P-value Final.Odds.Ratio       95% CI
CCP           0.154            1.694 (1.03, 2.77)
stage         0.087            3.141 (1.33, 7.64)
grade         0.360
bcg           0.207
chemo         0.274
age           0.454
gender        0.589
       Final.P-value
CCP            0.036
stage           0.01
grade
bcg
chemo
age
gender
```

4 *R* functions for assessing the improvement in prognostic power when CCP score is added to the *best available* model

```
> ######################################################################
> ## various functions for calculating C-index (AUC),
> ## and IDI and NRI measurements to compare models
> ######################################################################
>
> library(Hmisc)
> library(survIDINRI)
> library(survC1)
> library(pROC)
> C.delta.logistic <- function(x1,x2,Y) {
+    keep = !is.na(Y); Y = Y[keep]; x1 = x1[keep]; x2 = x2[keep]
+    p1 = predict.logistic(Y, data.frame(x1))
+    p2 = predict.logistic(Y, data.frame(x2))
```

```
+    rcorrp.cens(p1,p2,Y)
+ }
> ##########################################################################
> ## difference in C-indices at time tau using
> ## x and x[,-i] as the predictors
> ##########################################################################
> C.delta <- function(time, event, x, i, tau = NULL, itr = 1000, seed = NULL) {
+   if (max(event, na.rm=T) != 1 | min(event, na.rm=T) != 0) {
+         stop("event must be either 0 or 1...\n")
+   }
+   if (is.null(tau)) tau = median(time, na.rm=T)
+   mydata = cbind(time, event)
+
+   keep = apply(is.na(mydata), 1, sum) ==0 &
+       apply(is.na(x), 1, sum) ==0
+
+   mydata = mydata[keep,]
+   x = x[keep,]
+
+   covs0 = x[,-i]
+   covs1 = x
+   II = Inf.Cval.Delta(mydata,covs0, covs1, tau, itr = itr, seed = seed)
+   return(II)
+ }
> no.na <- function(x) apply(is.na(x),1,sum) ==0
> ####################################################
> ## predicts cox score
> ## if loocv is TRUE, then loocv is used and samples
> ##    are classified as high or low risk
> ####################################################
> predict.cox <- function(time, event, d, loocv = FALSE) {
+   keep =  no.na(cbind(time, event, d))
+   time = time[keep]; event = event[keep]
+   d = d[keep,]
+   if (is.null(nrow(d))) d = data.frame(d)
+
+   if (!loocv) {
+     S = Surv(time, event)
+     if (ncol(d) == 1) {
+         fit = coxph(S ~ d[,1])
```

```
+     } else {
+          fit = coxph(S ~ ., data = d[,])
+     }
+     p = predict(fit)
+     return(p)
+   }
+   p = LOO.risk(time, event, t(d), 0.50)
+   return(p)
+ }
> #####################################################
> ## predicts probability using logistic regression
> ##    y = responses (must be 0 or 1)
> ##    d = data.frame of predictors
> #####################################################
> predict.logistic <- function(y, d, loocv = FALSE) {
+   keep =  no.na(cbind(y, d))
+   y = y[keep]; d = d[keep,]
+   if (is.null(nrow(d))) d = data.frame(d)
+   if (!loocv) {
+     g = glm(y ~ ., data = d, family = "binomial")
+     p = predict(g, type = "response")
+     return(p)
+   }
+   p = rep(NA, nrow(d))
+   for (i in 1:length(p)) {
+     g = glm(y ~ ., data = d, family = "binomial", subset = -i)
+     newdata = d[i,]
+     if (ncol(d)==1) {
+         newdata = data.frame(newdata)
+         colnames(newdata) = colnames(d)
+     }
+     p[i] = predict(g, type = "response", newdata)
+   }
+   return(p)
+ }
> ######################################################
> ## C-index (AUC) from cox proportional hazards model
> ##   or logistic regression
> ## d = data.frame of predictor variables used
> ## time = time or NULL for classification
```

```
> ## event = 0 or 1
> ######################################################
> C.index <- function (time, event, d, loocv = FALSE) {
+   if (is.null(time)) {
+           keep = !is.na(event) & apply(is.na(d),1,sum) == 0
+         S = event[keep]
+   } else {
+           keep = !is.na(time) & !is.na(event) & apply(is.na(d),1,sum) == 0
+           S = Surv(time[keep], event[keep])
+   }
+
+   if (is.null(time))
+         p = predict.logistic(event, d, loocv)
+   else  p = -predict.cox(time, event, d, loocv)
+   r <- rcorr.cens(p,S)
+   return(r)
+ }
> is.right.range <- function(x, a = 0, b = 1) {
+   m1 = min(x, na.rm=T)
+   m2 = max(x, na.rm=T)
+   if (m1 == a & m2 == b) return(TRUE)
+   return(FALSE)
+ }
> ###########################################################################
> ## C-statistic for combinations of columns of d
> ## all columns are used + combs which should be a list
> ## ex: combs = list(NULL); combs[[1]] = 1:2; combs[[2]] = 1:3
> ##     will use columns (1,2) and (1,2,3) as predictors
> ###########################################################################
> C.index.by.col <- function(time, event, d, combs = NULL, loocv = FALSE) {
+   if (!is.right.range(event)) stop ("event must be in (0,1) ...\n")
+
+   if (is.null(time)) {
+         keep = !is.na(event) & apply(is.na(d),1,sum) == 0
+   } else {
+         keep = !is.na(time) & !is.na(event) & apply(is.na(d),1,sum) == 0
+           time = time[keep]
+   }
+   event = event[keep]; d = d[keep,]
+   ans = NULL
```

```
+   for (i in 1:ncol(d)) {
+     ans = rbind(ans, C.index(time, event, data.frame(d[,i]), loocv))
+   }
+   rownames(ans) = colnames(d)
+   if (is.null(combs)) return(ans)
+   ans.combs = NULL
+   for (i in 1:length(combs)) {
+     ans.combs = rbind(ans.combs,
+                       C.index(time, event, data.frame(d[,combs[[i]]]), loocv))
+   }
+   r = lapply(combs, function(x, r) paste(r[x], collapse = ":"), colnames(d))
+   r = unlist(r)
+   rownames(ans.combs) = r
+
+   ans = rbind(ans, ans.combs)
+   return(ans)
+ }
> ########################################################################
> ## calculates IDI at time t0 for x vs. x[,-i]
> ########################################################################
> IDI <- function(time, event, x, i, t0 = NULL, npert = 1000, seed1 = NULL) {
+   indata = cbind(time, event)
+   covs0 = x[,-i]
+   covs1 = x
+   keep = no.na(cbind(indata,covs1))
+   ii = IDI.INF(indata[keep,], covs0[keep,], covs1[keep,],
+                t0, npert = npert, seed1 = seed1)
+   return(IDI.INF.OUT(ii))
+ }
> format.ci <- function(x, digits = 2) {
+   x = round(x, digits)
+   x = paste("(", paste(x, collapse = ","), ")", sep = "")
+   return(x)
+ }
> ############################################################
> ## for models consisting of predictors x vs. x[,-i],
> ## calculates IDI, NRI, C, and delta C statistics
> ############################################################
> nested.improve.cox <- function(time, event, x, i, t0, npert,
+                                seed = NULL, loocv = FALSE) {
```

```
+     indata = cbind(time, event)
+     covs0 = x[,-i]
+     if (is.null(nrow(covs0))) covs0 = data.frame(covs0)
+     covs1 = x
+     keep = no.na(cbind(indata, covs1))
+
+     indata = indata[keep,]; time = time[keep]; event = event[keep]
+     covs0 = covs0[keep,]
+     if (is.null(nrow(covs0))) covs0 = data.frame(covs0)
+     covs1 = covs1[keep,]
+
+     p0 = covs0; p1 = covs1
+
+     if (loocv) {
+          p0 = predict.cox(time, event, covs0, loocv)
+          p1 = predict.cox(time, event, covs1, loocv)
+          p0 = data.frame(p0); p1 = data.frame(p1)
+     }
+
+     deltaC = Inf.Cval.Delta(indata,p0, p1, t0, itr = npert, seed = seed)
+     ii = IDI.INF(indata, p0, p1, t0, npert = npert, seed1 = seed)
+     ii = rbind(M1 = ii$m1, M2 = ii$m2, M3 = ii$m3)
+
+     combs = list(NULL)
+     combs[[1]] = (1:ncol(x))[-i]
+     combs[[2]] = 1:ncol(x)
+
+     CC = C.index.by.col(indata[,1], indata[,2], covs1, combs = combs,
+                         loocv = loocv)
+     CC = CC[,'C Index']
+     CC = cbind(CC)
+    digits = 2
+    CC = round(CC, digits)
+
+    ii.ci = apply(ii[,2:3], 1, format.ci, digits = digits)
+    ii = round(ii[,1], digits)
+
+     Ct = round(deltaC[1:2,'Est'], digits)
+     dCt = round(deltaC[3,1], digits)
+     dCt.ci = format.ci(deltaC[3,3:4], digits)
```

```
+
+    m0 = coxph(Surv(time, event) ~ ., data = covs0)
+    m1 = coxph(Surv(time, event) ~ ., data = covs1)
+    aa = anova(m0, m1, test = "Chisq")
+    P = aa$"P(>|Chi|)"[2]
+
+    names(Ct) = NULL
+    nn = rep(NA, nrow(CC))
+    ans = c(Ct = Ct[1], dCt = dCt, dCt.ci = dCt.ci, M1 = ii[1],
+                 M1.ci = ii.ci[1], M2 = ii[2], M2.ci = ii.ci[2],
+                 M3 = ii[3], M3.ci = ii.ci[3], P = round(P, 3))
+    rownames(ans) = NULL
+    nn = rep(NA, nrow(CC))
+    CC = cbind(CC, Ct = nn, dCt = nn, dCt.ci = nn, M1 = nn,
+               M1.ci = nn, M2 = nn, M2.ci = nn, M3 = nn, M3.ci = nn, P = nn)
+    CC[nrow(CC),2:11] = ans
+    CC[nrow(CC)-1, 2] = Ct[2]
+    return(CC)
+ }
> ###########################################################
> ## for models consisting of predictors x vs. x[,-i],
> ## calculates IDI, NRI, C, and delta C statistics
> ###########################################################
> nested.improve.logistic <- function(y, x, i, loocv = FALSE) {
+    keep = no.na(cbind(y,x))
+    x = x[keep,]
+    y = y[keep]
+    x0 = x[,-i]
+    if (is.null(nrow(x0))) {
+         x0 = data.frame(x0)
+         colnames(x0) = colnames(x)[-i]
+    }
+
+    m0 = glm(y ~ ., data = x0, family = "binomial")
+    m1 = glm(y ~ ., data = x, family = "binomial")
+
+    aa = anova(m0, m1, test = "Chisq")
+    P = aa$"Pr(>Chi)"[2]
+
+    p1 = predict.logistic(y,x0,loocv)
```

```
+    p2 = predict.logistic(y,x, loocv)
+
+    combs = list(NULL)
+    combs[[1]] = (1:ncol(x))[-i]
+    combs[[2]] = 1:ncol(x)
+
+    CC = C.index.by.col(NULL, y, x, combs = combs, loocv = loocv)
+    CC = CC[,'C Index']
+    CC = cbind(CC)
+
+    ###########################################################
+    ## construct CI for difference in AUCs using Delong's sd ##
+    ###########################################################
+    rr = roc.test(y, p1, p2)
+    s = abs(diff(rr$estimate) / rr$statistic)
+    c.delta = diff(rr$estimate)
+    rr.ci = c.delta + c(-1,1)* qnorm(.975)*s
+
+    ii = improveProb(p1, p2, y)
+    nri.ci = ii$nri + c(-1,1) * qnorm(.975)*ii$se.nri
+    idi.ci = ii$idi + c(-1,1) * qnorm(.975)*ii$se.idi
+
+    nri.ci = format.ci(nri.ci)
+    idi.ci = format.ci(idi.ci)
+    rr.ci = format.ci(rr.ci)
+
+    digits = 2
+    c.delta = round(c.delta, digits)
+    ii$nri = round(ii$nri, digits)
+    ii$idi = round(ii$idi, digits)
+    CC = round(CC,digits)
+
+    ans = c(c.delta = c.delta, c.delta.ci = rr.ci, nri = ii$nri,
+            nri.ci = nri.ci, idi = ii$idi, idi.ci = idi.ci, P = round(P, 3))
+    rownames(ans) = NULL
+    nn = rep(NA, nrow(CC))
+    CC = cbind(CC, c.delta = nn, c.delta.ci = nn, nri = nn,
+               nri.ci = nn, idi = nn, idi.ci = nn, P = nn)
+    CC[nrow(CC),2:8] = ans
+    return(CC)
```

```
+ }
>
>
>
```

5 Assessing the improvement in prognostic power when CCP score is added to the *best available* progression and survival models for CNUH

```
> ########################################################################
> ### This code evaluates the ability of the CCP gene signature to
> ### discriminate between high and low risk patients,
> ### and tests for added value beyond clinical variables
> ########################################################################
>
>
> TAU = 60
> library(survIDINRI)
> library(ROCR)
> library(survival)
> ####################################################################
> ## Note: p-values may differ slightly from published table do to ##
> ## a different random number seed being used                     ##
> ####################################################################
>
> ###################################################################
> ## Added value of CCP score to BA model for progression in CNUH ##
> ###################################################################
> data.korea = data.frame(CCP = korea.score, stage = data.korea$stage)
> cc.p.korea = nested.improve.logistic(korea.progression-1, data.korea, 1, FALSE)
> cc.p.korea[is.na(cc.p.korea)] = ""
> cc.p.korea

          CC     c.delta c.delta.ci nri   nri.ci
CCP       "0.68" ""      ""         ""    ""
stage     "0.67" ""      ""         ""    ""
stage     "0.67" ""      ""         ""    ""
CCP:stage "0.73" "0.06"  "(0,0.12)" "0.5" "(0.12,0.87)"
          idi    idi.ci     P
```

```
CCP       ""     ""          ""
stage     ""     ""          ""
stage     ""     ""          ""
CCP:stage "0.03" "(0,0.05)" "0.036"
```

```
> ##################################################################
> ## Added value of CCP score to BA model for survival in CNUH    ##
> ##################################################################
> data.korea = data.frame(CCP = data.korea$CCP, stage = data.korea$stage,
+                         age = GSE13507.age)
> cc.s.korea = nested.improve.cox(GSE13507.DSS.time, GSE13507.DSS.outcome,
+                                 data.korea, 1, TAU, 1000)
> cc.s.korea[is.na(cc.s.korea)] = ""
> cc.s.korea
```

```
              CC     Ct     dCt    dCt.ci          M1
CCP           "0.72" ""     ""     ""              ""
stage         "0.8"  ""     ""     ""              ""
age           "0.68" ""     ""     ""              ""
stage:age     "0.87" "0.87" ""     ""              ""
CCP:stage:age "0.88" "0.88" "0.01" "(-0.02,0.03)" "0"
              M1.ci          M2     M2.ci          M3
CCP           ""             ""     ""             ""
stage         ""             ""     ""             ""
age           ""             ""     ""             ""
stage:age     ""             ""     ""             ""
CCP:stage:age "(-0.01,0.07)" "0.15" "(-0.37,0.39)" "0.01"
              M3.ci          P
CCP           ""             ""
stage         ""             ""
age           ""             ""
stage:age     ""             ""
CCP:stage:age "(-0.01,0.08)" "0.237"
```

6 R function for adjusting gene expression data by a specified score (e.g., CCP)

```
> ##############################################################
> ## adjusts the expression value of each gene by 'pc' if     #
> ## intercept.only is FALSE; otherwise the expression values #
```

```
> ## are adjusted by a constant                              #
> ############################################################
> global.adjust <- function(X, pc, intercept.only = FALSE) {
+   newX = NULL
+   if (!intercept.only) {
+     newX = apply(X, 1, function(x,pc) { l = lm(x~pc); return(l$residuals) }, pc[,1]
+   } else {
+     newX = apply(X, 1, function(x) { l = lm(x~1); return(l$residuals) })
+   }
+   newX = t(newX)
+   rownames(newX) = rownames(X)
+   return(newX)
+ }
```
