# Supplementary material for: Robust Prognostic Gene Expression Signatures in Bladder Cancer and Lung Adenocarcinoma Depend on Cell Cycle Related Genes
Source: PLoS One. 2014 Jan 22;9(1):e85249. doi: 10.1371/journal.pone.0085249 (PMC3898982; doi:10.1371/journal.pone.0085249)
Supplement: File S4 — Multivariate analyses of progression in bladder cancer (Table S6), survival in bladder cancer (Table S7), and survival in lung adenocarcinoma (Table S8). (PDF) [file pone.0085249.s004.pdf]

Supplementary Table S6. Multivariate analysis of progression in bladder cancer

Dataset (*N*)	Variable	Univariate analysis Odds Ratio (95% CI)	P	Multivariate analysis Odds Ratio (95% CI)	P	Final model[†] Odds Ratio (95% CI)	P
**CNUH (165)**	**CCP score***	2.08 (1.29, 3.38)	0.003	1.52 (0.84, 2.76)	0.164	1.7 (1.02, 2.83)	0.041
	**Stage (Ta-T1 vs. T2-T4)**[ǁ]	4.12 (1.81, 9.39)	0.001	3.58 (0.84, 15.29)	0.085	3.19 (1.33, 7.64)	0.009
	High grade vs. low grade	3.59 (1.6, 8.08)	0.002	1.63 (0.6, 4.44)	0.342		
	BCG	0.62 (0.26, 1.5)	0.291	2.47 (0.61, 10.05)	0.207		
	Chemotherapy vs. no treatment	3.28 (1.32, 8.13)	0.01	1.93 (0.6, 6.23)	0.273		
	Age	1.02 (0.99, 1.06)	0.173	1.02 (0.97, 1.06)	0.456		
	Male vs. Female	0.91 (0.34, 2.46)	0.851	1.31 (0.47, 3.68)	0.604		
**Lindgren (97)**	**CCP score***	2.65 (1.24, 5.66)	0.012	2.04 (0.76, 5.46)	0.157	2.65 (1.24, 5.66)	0.012
	High grade vs. low grade	3.79 (1.22, 11.81)	0.022	2.06 (0.44, 9.58)	0.358		
	**Variable**	**Hazard Ratio (95% CI)**	**P**	**Hazard Ratio (95% CI)**	**P**	**Hazard Ratio (95% CI)**	**P**
**Dyrskjot (155)**	**CCP score***	5.18 (2.91, 9.25)	<0.001	3.95 (1.79, 8.72)	0.001	5.18 (2.91, 9.25)	< 0.001
	Stage (T1 vs. Ta)	2.32 (1.17, 4.63)	0.017	1.01 (0.38, 2.69)	0.988		
	High grade vs. low grade	2.53 (1.11, 5.76)	0.027	1.38 (0.43, 4.44)	0.59		
	PUNLMP vs. low grade	0.36 (0.05, 2.45)	0.297	0.83 (0.1, 6.7)	0.863		
	CIS diagnosis	2.61 (1.31, 5.18)	0.006	1.76 (0.68, 4.55)	0.245		
	BCG/MMC vs. no treatment	0.89 (0.39, 2.01)	0.774	0.42 (0.13, 1.33)	0.139		
	Age	1.07 (1.03, 1.1)	<0.001	1.02 (0.98, 1.07)	0.264		
	Male vs. Female	0.8 (0.36, 1.75)	0.574	1.13 (0.43, 3.01)	0.801		

*CCP score is a continuous variable. Odds and hazard ratios are with respect to a 1-unit increase in CCP score

[ǁ]Stage is confounded with surgery in CNUH (all patients with invasive T2-T4 tumors received cystectomies and all patients with non-invasive Ta-T1 tumors received transurethral resections

[†]Final model is constructed from forward step-wise regression of significant variables (P < 0.05) and includes variables in **bold**

Abbreviations: PUNLMP, papillary urothelial neoplasm of low malignant potential; BCG, Bacillus Calmette-Guerin; MMC, mitomycin-C

Supplementary Table S7. Multivariate analysis of survival in bladder cancer

Dataset (*N*)		Univariate analysis Hazard ratio (95% CI)	P	Multivariate analysis Hazard ratio (95% CI)	P	Final model Hazard ratio (95% CI)	P
**Blaveri (78)**	**CCP score***	2.14 (1.32, 3.46)	0.002	2.06 (1.22, 3.5)	0.007	2.14 (1.32, 3.46)	0.002
	High grade vs. low grade	2.22 (0.79, 6.26)	0.132	0.97 (0.24, 3.96)	0.962		
	Stage (Ta-T1 vs. T2-T4)	1.85 (0.9, 3.78)	0.093	1.98 (0.51, 7.73)	0.326		
	Cystectomy vs. TURBT	1.48 (0.74, 2.93)	0.266	0.84 (0.22, 3.24)	0.795		
	Age	1 (0.97, 1.04)	0.804	1 (0.96, 1.03)	0.89		
	Male vs. female	1.09 (0.56, 2.13)	0.789	1.05 (0.48, 2.28)	0.901		
**CNUH (165)**	CCP score*	2.35 (1.48, 3.72)	<0.001	1.17 (0.69, 1.96)	0.563		
	**Stage (Ta-T1 vs. T2-T4)**[‖]	17.9 (6.44, 49.75)	<0.001	9.81 (1.84, 52.47)	0.008	18.26 (6.53, 51.03)	<0.001
	High grade vs. low grade	5.99 (2.82, 12.72)	<0.001	1.24 (0.49, 3.14)	0.643		
	BCG vs. no treatment	0.1 (0.03, 0.43)	0.002	0.69 (0.09, 5.49)	0.73		
	Chemotherapy vs. no treatment	4.25 (2.13, 8.49)	<0.001	1.84 (0.77, 4.37)	0.169		
	**Age**	1.05 (1.02, 1.08)	0.002	1.06 (1.02, 1.11)	0.008	1.06 (1.02, 1.1)	0.003
	Male vs. female	0.48 (0.22, 1.04)	0.063	0.65 (0.27, 1.56)	0.334		
**Dyrskjot (155)**	**CCP score***	3.99 (1.93, 8.23)	<0.001	2.73 (1.21, 6.15)	0.015	2.3 (1.07, 4.94)	0.033
	Stage (Ta-T1 vs. T2-T4)[#]	0 (0, Inf)	0.997	0 (0, Inf)	0.998		
	High grade vs. low grade	1.73 (0.74, 4.08)	0.207	0.85 (0.31, 2.35)	0.751		
	PUNLMP vs. low grade	0.44 (0.06, 3.23)	0.418	1.12 (0.13, 9.76)	0.919		
	**CIS diagnosis**	3.38 (1.6, 7.16)	0.001	2.78 (1.16, 6.64)	0.021	1.98 (0.88, 4.41)	0.097
	Cystectomy after TURBT	0 (0, Inf)	0.997	0 (0, Inf)	0.998		
	BCG/MMC vs. no treatment	1.13 (0.46, 2.78)	0.796	0.55 (0.2, 1.48)	0.234		
	**Age**	1.09 (1.04, 1.14)	0.001	1.05 (1, 1.1)	0.062	1.06 (1.01, 1.11)	0.015
	Male vs. female	1.3 (0.49, 3.42)	0.593	1.24 (0.46, 3.31)	0.671		
**Lindgren (156)**	CCP score*	2.84 (1.68, 4.82)	<0.001	1.3 (0.6, 2.85)	0.508		
	High grade vs. low grade	6.62 (2.28, 19.21)	0.001	0.97 (0.13, 7.17)	0.972		
	**Stage (Ta-T1 vs. T2-T4)**	12.82 (5.03, 32.7)	<0.001	19.93 (2.16, 183.66)	0.008	12.82 (5.03, 32.7)	<0.001
	Cystectomy after TURBT	2.93 (1.33, 6.45)	0.007	0.4 (0.13, 1.25)	0.114		
**MSKCC (87)**	CCP score*	1.81 (1.09, 3)	0.022	1.77 (1, 3.12)	0.048		
	**High grade vs. low grade**	11.29 (1.8, 70.71)	0.01	6.17 (1.8, 21.24)	0.004	5.31 (0.98, 28.69)	0.053
	**Stage (Ta-T1 vs. T2-T4)**	14.61 (2.13, 100.22)	0.006	10.22 (1.64, 63.67)	0.013	8.74 (1.24, 61.56)	0.03
	Age	1 (0.96, 1.04)	0.904	1.01 (0.98, 1.05)	0.505		
	Male vs. female	1.5 (0.68, 3.31)	0.32	1.57 (0.66, 3.74)	0.304		

*CCP score is a continuous variable. Hazard ratios are with respect to a 1-unit increase in CCP score

[‖]Stage is confounded with surgery in CNUH (all patients with invasive T2-T4 tumors received cystectomies and all patients with non-invasive Ta-T1 tumors received transurethral resections

[#]Stage in Dyrskjot has a hazard ratio of 0 due to the presence of five patients with T2-T4 tumors that are all censored

[†]Final model is constructed from forward step-wise regression of significant variables (P < 0.05) and includes variables in **bold**

Abbreviations: DSS, disease-specific survival; OS, overall survival; TURBT, transurethral resection of the bladder; PUNLMP, papillary urothelial neoplasm of low malignant potential; BCG, Bacillus Calmette-Guerin; MMC, mitomycin-C

Supplementary Table S8. Multivariate analysis of survival in lung adenocarcinoma

Dataset (endpoint, N)	Variable	Univariate analysis Hazard Ratio (95% CI)	P	Multivariate analysis Hazard Ratio (95% CI)	P	Final model† Hazard Ratio (95% CI)	P
**CAN/DF (OS, N = 73)**	**CCP score***	1.73 (1.07, 2.79)	0.024	1.91 (1.1, 3.32)	0.021	1.92 (1.13, 3.26)	0.017
	Stage I vs. II	0.3 (0.15, 0.59)	<0.001	0.38 (0.17, 0.87)	0.023	0.39 (0.18, 0.86)	0.019
	Poorly vs. moderately differentiated	1.45 (.73, 2.90)	0.291	0.94 (.36, 2.5)	0.905		
	Well vs. moderately differentiated	0.98 (0.4, 2.4)	0.957	1.19 (0.46, 3.1)	0.721		
	Current/former smoker vs. never	0.52 (0.18, 1.51)	0.226	0.71 (0.24, 2.13)	0.543		
	**Chemotherapy vs. no treatment**	1.46 (0.74, 2.86)	0.272	2.78 (1.21, 6.36)	0.016	2.31 (1.09, 4.88)	0.028
	**Age**	1.08 (1.04, 1.13)	<0.001	1.08 (1.03, 1.13)	0.001	1.09 (1.04, 1.13)	<0.001
	Male vs. female	1.77 (0.84, 3.71)	0.133	1.64 (0.57, 4.7)	0.359		
**MKS (OS, N = 98)**	CCP score*	1.65 (1.14, 2.37)	0.008	1.58 (0.98, 2.56)	0.062		
	Stage III vs. II	3.52 (1.75, 7.08)	<0.001	1.24 (0.3, 5.2)	0.764		
	Stage I vs. II	0.34 (0.18, 0.66)	0.001	0.57 (0.16, 1.95)	0.368		
	**Poorly vs. moderately differentiated**	3.75 (1.83, 7.68)	<0.001	3.3 (1.28, 8.53)	0.014	3.49 (1.77, 6.87)	<0.001
	Well vs. moderately differentiated	0.75 (0.28, 2.02)	0.573	4.56 (1.25, 16.61)	0.021		
	Radiotherapy vs. no treatment	3.66 (1.93, 6.95)	<0.001	1.48 (0.53, 4.16)	0.452		
	**Chemotherapy vs. no treatment**	3.93 (2.04, 7.55)	<0.001	2.53 (0.91, 7.05)	0.077	3.65 (1.83, 7.29)	<0.001
	Current/former smoker vs. never	1.13 (0.46, 2.79)	0.783	0.8 (0.31, 2.05)	0.642		
	Age	0.99 (0.96, 1.03)	0.65	0.99 (0.96, 1.03)	0.760		
	Male vs. female	0.97 (0.49, 1.92)	0.923	1.12 (0.49, 2.55)	0.786		
**Takeuchi (OS, N = 90)**	**CCP score***	2.68 (1.54, 4.67)	0.001	2.58 (1.28, 5.2)	0.008	2.67 (1.49, 4.79)	<0.001
	Poorly vs. moderately differentiated	1.94 (1.04, 3.61)	0.037	1.53 (0.63, 3.7)	0.351		
	Well vs. moderately differentiated	0.63 (0.31, 1.28)	0.204	1.25 (0.55, 2.85)	0.594		
	Stage III vs. II	3.01 (1.62, 5.57)	<0.001	1.5 (0.5, 4.51)	0.467		
	**Stage I vs. II**	0.37 (0.2, 0.69)	0.002	0.53 (0.19, 1.42)	0.205	0.38 (0.2, 0.72)	<0.001
	EGFR (mut vs. WT)	0.94 (0.69, 1.29)	0.701	0.74 (0.52, 1.07)	0.112		
	KRAS (mut vs. WT)	0.75 (0.48, 1.18)	0.208	0.77 (0.42, 1.4)	0.389		
	p53 (mut vs. WT)	0.93 (0.68, 1.28)	0.667	1.03 (0.7, 1.53)	0.879		
	Current/former smoker vs. never	1.53 (0.83, 2.82)	0.171	0.98 (0.39, 2.45)	0.960		
	Age	1 (0.97, 1.04)	0.798	1.01 (0.97, 1.04)	0.776		
	Male vs. female	1.33 (0.72, 2.45)	0.356	1.2 (0.41, 3.49)	0.733		
**Tomida (OS, N = 116)**	**CCP score***	1.87 (1.29, 2.71)	0.001	1.99 (1.25, 3.18)	0.004	1.81 (1.23, 2.68)	0.003
	**Stage III vs. II**	2.94 (1.62, 5.32)	<0.001	2.21 (0.8, 6.1)	0.127	2.68 (1.42, 5.04)	0.002
	Stage I vs. II	0.4 (0.23, 0.7)	0.001	0.73 (0.3, 1.78)	0.486		
	EGFR (mut vs. WT)	1 (0.57, 1.76)	0.989	0.63 (0.29, 1.36)	0.237		
	KRAS (mut vs. WT)	0.66 (0.31, 1.41)	0.285	0.68 (0.26, 1.76)	0.427		
	p53 (mut vs. WT)	0.86 (0.65, 1.15)	0.311	1.05 (0.75, 1.48)	0.774		
	Current/former smoker vs. never	1.34 (0.77, 2.32)	0.303	0.63 (0.26, 1.55)	0.318		
	Age	1.01 (0.98, 1.03)	0.703	1.01 (0.98, 1.04)	0.654		
	Male vs. female	1.33 (0.77, 2.32)	0.307	1.6 (0.61, 4.2)	0.337		

*CCP score is a continuous variable. Hazard ratios are with respect to a 1-unit increase in CCP score

†Final model is constructed from forward step-wise regression of significant variables ($P < 0.05$) and includes variables in **bold**

Abbreviations: OS, overall survival; mut, mutant; WT, wild-type
