# Supplementary material for: Robust Prognostic Gene Expression Signatures in Bladder Cancer and Lung Adenocarcinoma Depend on Cell Cycle Related Genes
Source: PLoS One. 2014 Jan 22;9(1):e85249. doi: 10.1371/journal.pone.0085249 (PMC3898982; doi:10.1371/journal.pone.0085249)
Supplement: File S5 — Comparison of prognostic power of CCP score and best available clinical variables in bladder (Table S9) and lung adenocarcinoma (Table S10). (PDF) [file pone.0085249.s005.pdf]

Supplementary Table S9. Comparison of prognostic power of CCP score and best available clinical variables in bladder cancer

		Best available model		+CCP score	Improvement		
Dataset	Endpoint	Clinical variables*	C	C	ΔC	IDI	P-value
**CNUH (N=165)**	Progression	Stage	0.67	0.73	0.06	0.02	0.039
**Lindgren (N = 97)**	Progression	Grade	0.65	0.73	0.08	0.02	0.124
**Dyrskjot (N = 162)**	PFS	CIS, age	0.70	0.78	0.09	0.05	0.002
**Blaveri (N = 78)**	OS	Stage†	0.58	0.67	0.09	0.10	0.009
**CNUH (N = 165)**	DSS	Stage, age	0.87	0.88	0.01	0.00	0.262
**Dyrskjot (N = 155)**	DSS	CIS, age	0.74	0.78	0.04	0.02	0.061
**Lindgren (N = 156)**	DSS	Stage	0.79	0.82	0.03	0.00	0.347
**MSKCC (N = 87)**	OS	Stage, grade	0.68	0.71	0.03	0.06	0.070

*Significant variables selected from **Table 1** (excluding CCP score) by forward step-wise selection
Stage: Ta-T1 vs. T2-T4 (CNUH, Blaveri, Lindgren, and MSKCC)
Grade: high vs. low (CNUH, Lindgren, Blaveri, MSKCC)
†Included as best predictive clinical variable even though not significant in univariate analysis.
Abbreviations: PFS, progression-free survival; OS, overall survival; DSS, disease-specific survival; C, C-index; IDI, integrated discrimination improvement

Supplementary Table S10. Comparison of prognostic power of CCP score and best available clinical variables in lung adenocarcinoma

		Best available model		+CCP score	Improvement		
Dataset	Endpoint	Clinical variables*	C	C	ΔC	IDI	P-value
**CANDF**	OS	Stage, age	0.75	0.79	0.04	0.06	0.017
**MKS**	OS	Chemo, grade	0.74	0.75	0.01	0.00	0.185
**Takeuchi**	OS	Stage	0.63	0.73	0.10	0.07	0.004
**Tomida**	OS	Stage	0.61	0.71	0.07	0.07	0.001

*Significant variables selected from **Table 2** (excluding CCP score) by forward step-wise selection.
Stage: I vs. II or III (CANDF), III vs. I or II (Takeuchi, Tomida)
Grade: poorly vs. well or moderately differentiated
Abbreviations: OS, overall survival; C, C-index; IDI, integrated discrimination improvement
