# Supplementary material for: Robust Prognostic Gene Expression Signatures in Bladder Cancer and Lung Adenocarcinoma Depend on Cell Cycle Related Genes
Source: PLoS One. 2014 Jan 22;9(1):e85249. doi: 10.1371/journal.pone.0085249 (PMC3898982; doi:10.1371/journal.pone.0085249)
Supplement: File S8 — CCP genes in five refined signatures (Table S12). Signatures are denoted by the number of CCP genes. (DOCX) [file pone.0085249.s008.docx]

**Supplementary Table S12**. CCP genes in five refined signatures. Signatures are denoted by the number of CCP genes.

| **CCP-4** | **CCP-7** | **CCP-10** | **CCP-12** | **CCP-15** |
| --- | --- | --- | --- | --- |
| PTTG1 | PTTG1 | BUB1B | BIRC5 | ASF1B |
| BIRC5 | BIRC5 | CEP55 | BUB1B | ASPM |
| CDCA8 | TOP2A | DLGAP5 | CDC20 | C18orf24 |
| TK1 | CDCA8 | NUSAP1 | CDC2 | CDKN3 |
|  | KIAA0101 | PTTG1 | CDCA8 | CENPM |
|  | PRC1 | BIRC5 | CENPF | DLGAP5 |
|  | TK1 | CDCA8 | FOXM1 | DTL |
|  |  | KIAA0101 | KIF11 | KIAA0101 |
|  |  | PRC1 | NUSAP1 | KIF20A |
|  |  | TK1 | PTTG1 | MCM10 |
|  |  |  | TK1 | ORC6L |
|  |  |  | TOP2A | PRC1 |
|  |  |  |  | RAD51 |
|  |  |  |  | RAD54L |
|  |  |  |  | RRM2 |
