# Supplementary figures and images for: Robust Prognostic Gene Expression Signatures in Bladder Cancer and Lung Adenocarcinoma Depend on Cell Cycle Related Genes
Source: PLoS One. 2014 Jan 22;9(1):e85249. doi: 10.1371/journal.pone.0085249 (PMC3898982; doi:10.1371/journal.pone.0085249)

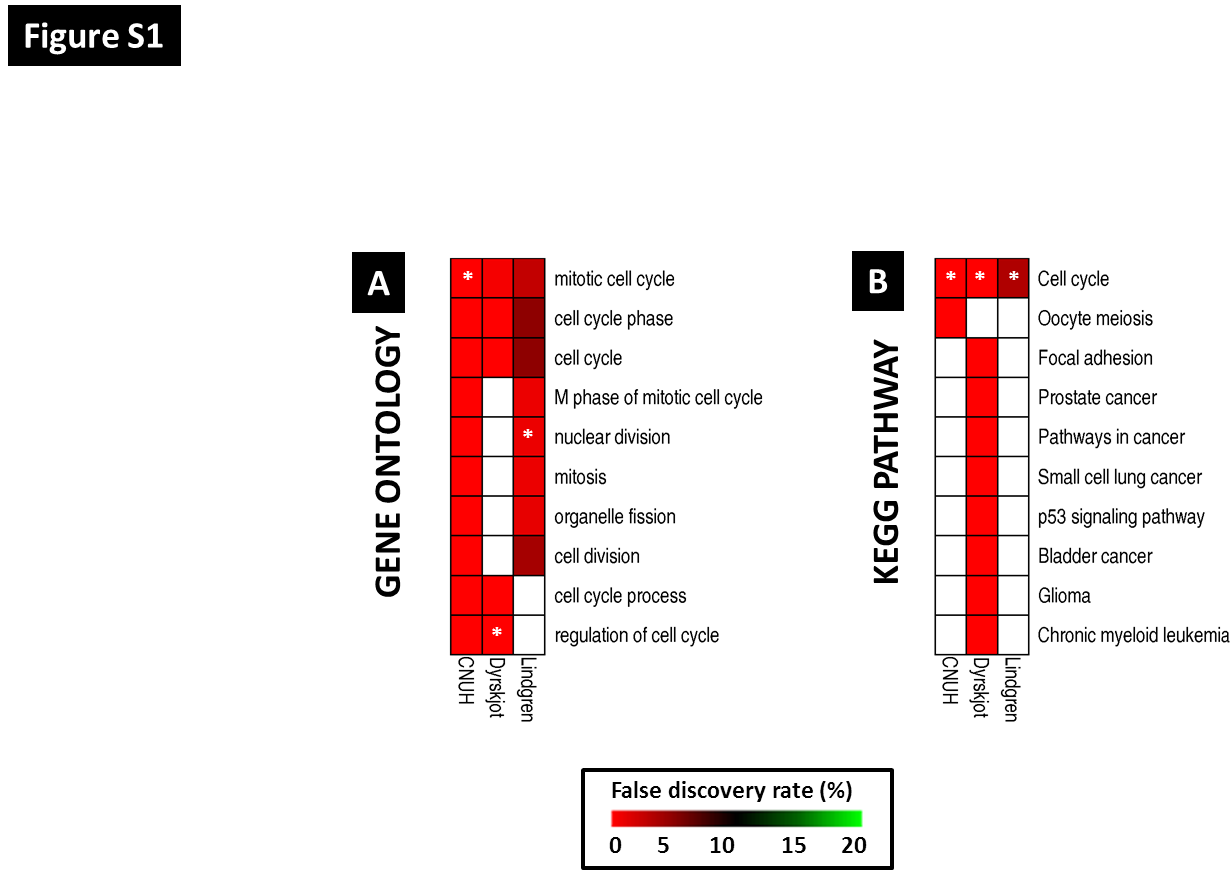

Supplement: Figure S1 — Prognostic modules associated with progression in bladder cancer. In each cohort, over-represented Gene Ontology (GO) terms and KEGG pathways were identified from lists of genes significantly predictive of progression (P<0.01) using the DAVID gene annotation enrichment analysis toolkit. Consistently prognostic modules were identified by ranking all modules first by the number of cohorts with significant results (FDR<20%) and then by average p-value. Each subfigure includes ten modules: the most consistently prognostic modules and the ‘top hit’ for each cohort, marked by an asterisk (*), which is defined as the module with the lowest false discovery rate (FDR) in that cohort that has an FDR<20% in multiple cohorts. A, over-represented GO terms associated with progression in bladder cancer. B, over-represented KEGG pathways associated with progression in bladder cancer. (TIF) [file pone.0085249.s010.tif]

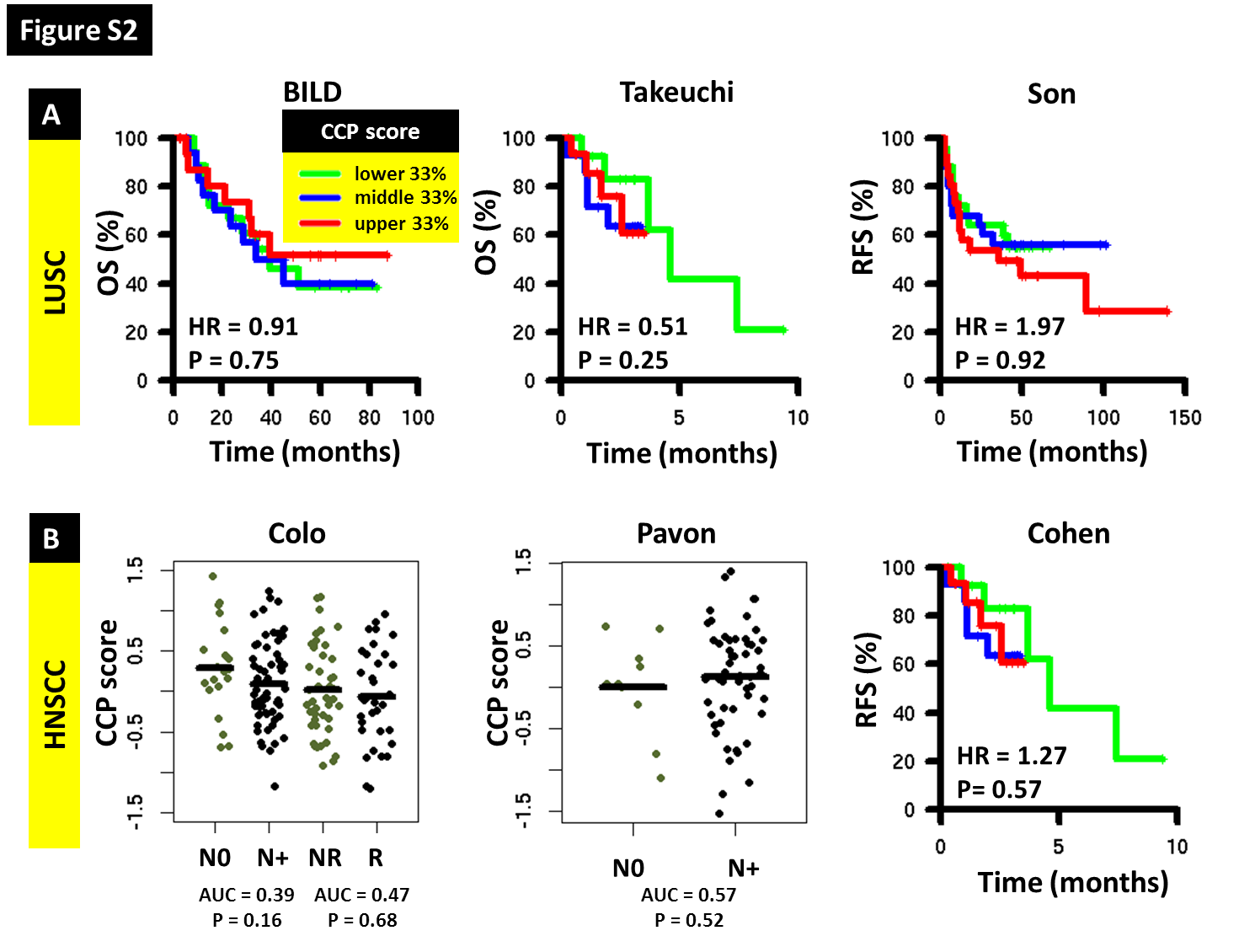

Supplement: Figure S2 — Prognostic value of CCP score in squamous cell lung cancers and head and neck squamous cell carcinomas. A, prognostic value of CCP score in squamous cell lung carcinomas (SCLC). Kaplan-Meier (KM) curves were generated for overall survival (OS) in the Bild (N = 53) and Takeuchi (N = 35) cohorts and for recurrence-free survival (RFS) in the Son (N = 76) cohort. KM curves were generated for patients with CCP scores at the lower (green), middle (blue), and upper (red) 33% and the log rank P-value of the continuous CCP score is reported. B, prognostic value of CCP score in head and neck squamous cell carcinomas (HNSCC). The Colo (N = 81) cohort did not include clinical follow-up time and so we evaluated the ability of CCP score to discriminate between node negative (N0) and node positive (N+) patients or between patients with non-recurrent (NR) and recurrent (R) tumors. The Pavon cohort (N = 63) did not include any clinical endpoints and so we evaluated the ability of CCP score to discriminate between N0 and N+ patients. In the Cohen cohort (N = 44), KM curves were generated for RFS. Abbreviations: HR, hazard ratio, corresponding to 1-unit increase in CCP score. (TIF) [file pone.0085249.s011.tif]

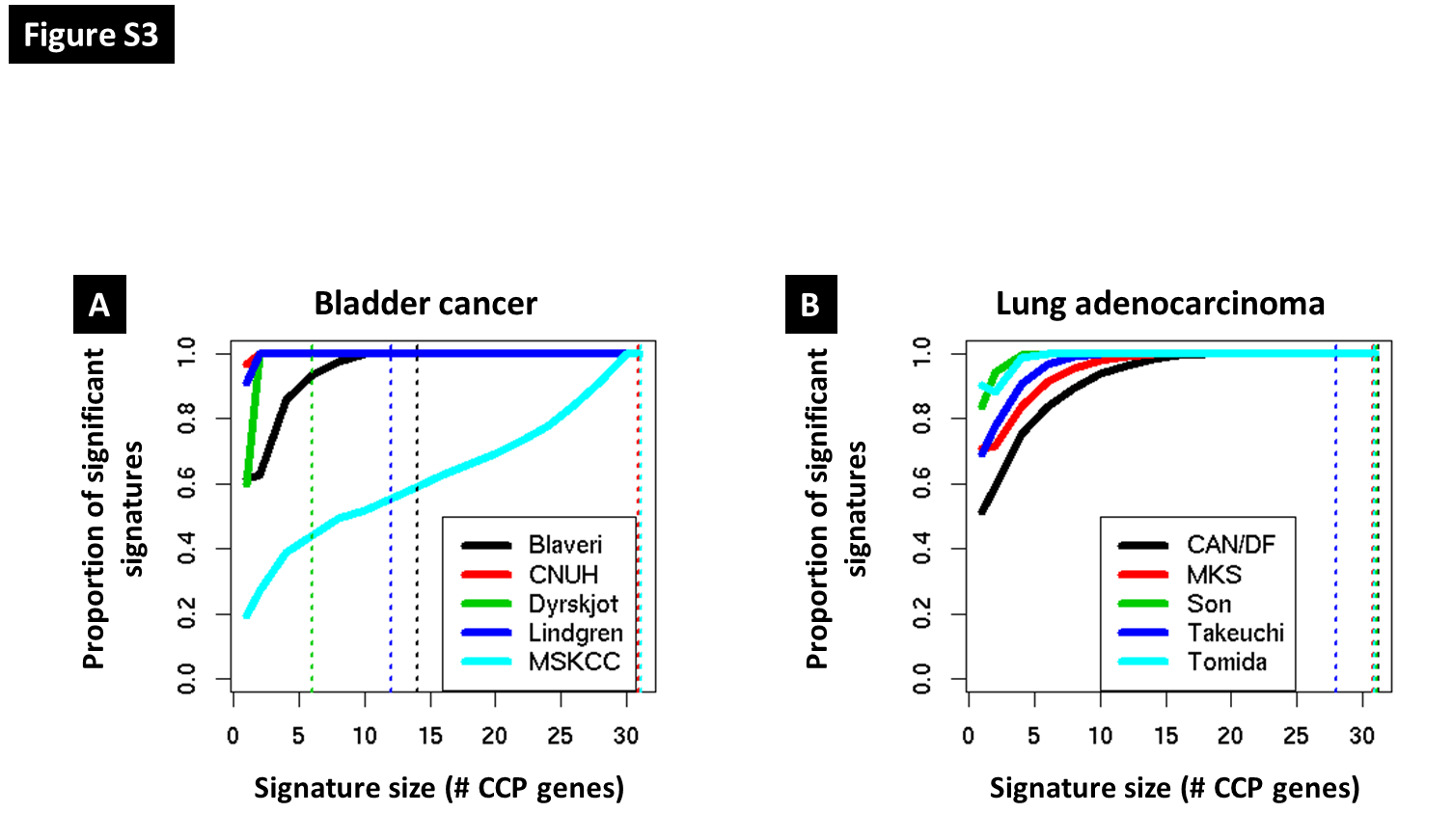

Supplement: Figure S3 — Relationship between prognostic value of CCP score and signature size based on proportion of significant signatures. Up to 10,000 gene signatures of sizes 1, 2, 4, …30, 31 were generated as described in Supporting Materials and Methods in File S1. Solid lines indicate proportion of signatures at each size that predicted survival (P<0.05) and are colored according to A, bladder patient cohort and B, lung adenocarcinoma cohort. Vertical dotted lines correspond to number of CCP genes (of 31) profiled in each cohort and are colored according to cohort. (TIF) [file pone.0085249.s012.tif]

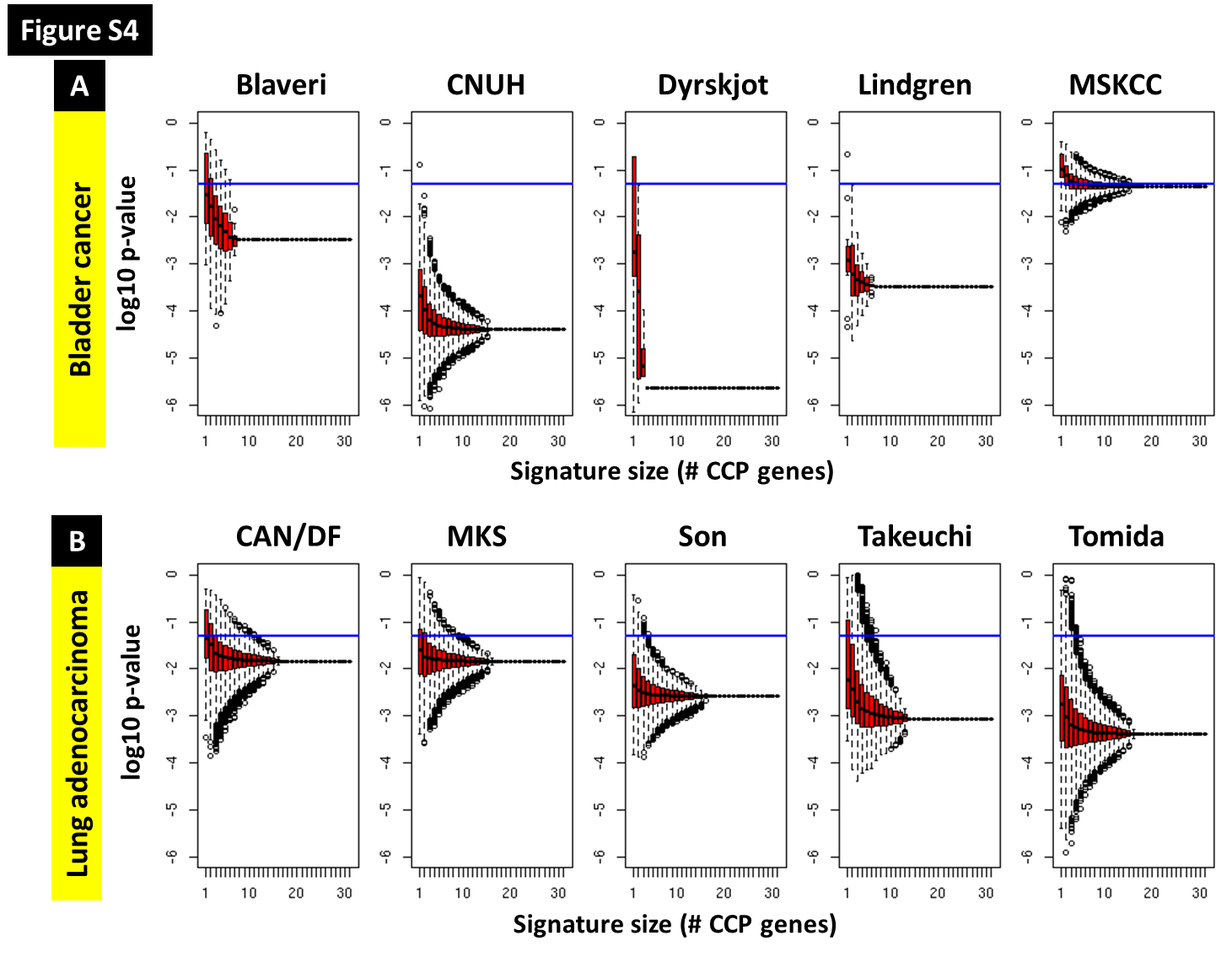

Supplement: Figure S4 — Relationship between prognostic value of CCP score and signature size based on p-values. Up to 10,000 gene signatures of sizes 1, 2, 4, …30, 31 were generated as described in Supporting Materials and Methods in File S1. Boxplots of log10 p-values of signature scores for each signature size are plotted in A, bladder patient cohorts and B, lung adenocarcinoma cohorts. The blue horizontal line corresponds to a p-value of 0.05. (TIF) [file pone.0085249.s013.tif]

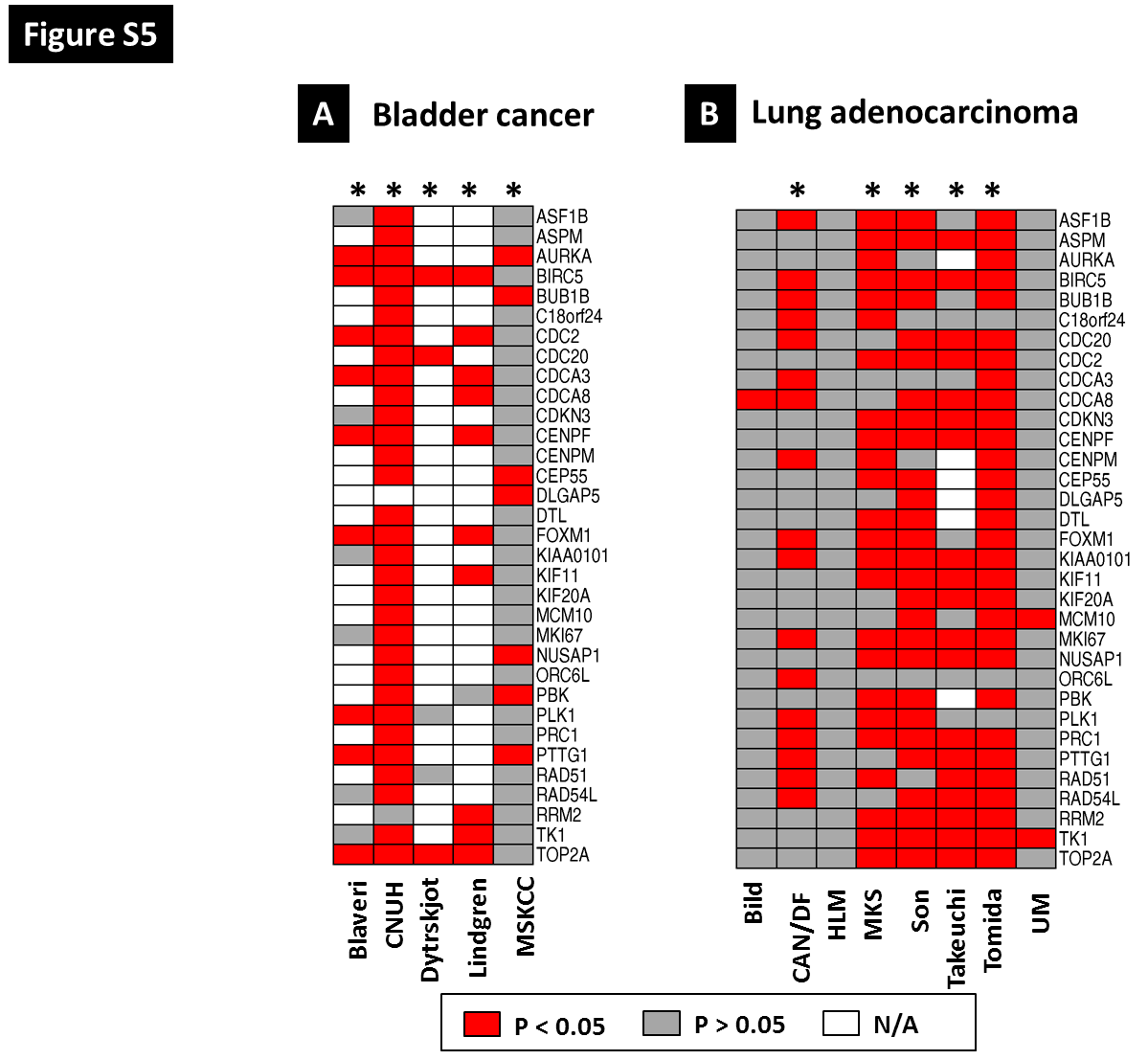

Supplement: Figure S5 — Prognostic value of CCP signature genes in A, bladder cancer and B, lung adenocarcinoma cohorts. In each cohort a gene is either significantly predictive of outcome (red box, P<0.05), not significantly predictive of outcome (gray box, P≥0.05), or was not profiled (white box) in each cohort. * indicates CCP score (using all available genes) is prognostic (P<0.05). (TIF) [file pone.0085249.s014.tif]
